# The Impact of Processing and Extraction Methods on the Allergenicity of Targeted Protein Quantification as Well as Bioactive Peptides Derived from Egg

**DOI:** 10.3390/molecules28062658

**Published:** 2023-03-15

**Authors:** Parisa Mostashari, Krystian Marszałek, Aynura Aliyeva, Amin Mousavi Khaneghah

**Affiliations:** 1 Department of Food Science and Technology, Faculty of Nutrition Sciences and Food Technology, National Nutrition and Food Technology Research Institute, Shahid Beheshti University of Medical Sciences, Tehran 1981619573, Iran; 2 Department of Food Science and Technology, Faculty of Pharmacy, Tehran Medical Sciences, Islamic Azad University, Tehran 1941933111, Iran; 3 Department of Fruit and Vegetable Product Technology, Prof. Wacław Dąbrowski Institute of Agricultural and Food Biotechnology, State Research Institute, 36 Rakowiecka St., 02-532 Warsaw, Poland; 4 Department of Technology of Chemistry, Azerbaijan State Oil and Industry University, 16/21 Azadliq Ave, AZ1010 Baku, Azerbaijan

**Keywords:** allergen, egg proteins, bioactive peptide, allergencity, processing methods, extraction methods

## Abstract

This review article discusses advanced extraction methods to enhance the functionality of egg-derived peptides while reducing their allergenicity. While eggs are considered a nutrient-dense food, some proteins can cause allergic reactions in susceptible individuals. Therefore, various methods have been developed to reduce the allergenicity of egg-derived proteins, such as enzymatic hydrolysis, heat treatment, and glycosylation. In addition to reducing allergenicity, advanced extraction methods can enhance the functionality of egg-derived peptides. Techniques such as membrane separation, chromatography, and electrodialysis can isolate and purify specific egg-derived peptides with desired functional properties, improving their bioactivity. Further, enzymatic hydrolysis can also break down polypeptide sequences and produce bioactive peptides with various health benefits. While liquid chromatography is the most commonly used method to obtain individual proteins for developing novel food products, several challenges are associated with optimizing extraction conditions to maximize functionality and allergenicity reduction. The article also highlights the challenges and future perspectives, including optimizing extraction conditions to maximize functionality and allergenicity reduction. The review concludes by highlighting the potential for future research in this area to improve the safety and efficacy of egg-derived peptides more broadly.

## 1. Introduction

Eggs are frequently considered one of nature’s most perfect products. They are consumed worldwide on a large scale without any religious or cultural restrictions, making them a popular and accessible dietary option. Laden with high nutritional value (including high-quality proteins and substantial amounts of vitamins, antioxidants, and other healthy compounds), distinctive fresh-like attributes, special functional characteristics (such as gelling, foaming, and emulsifying), and sensory quality, the use of egg fractions as a key ingredient has increased dramatically across various food industries (e.g., bakery products, noodles, powdered soup, desserts, snacks, confectionery, mayonnaise, sauces, and ice creams) [1,2]. The consumption of eggs in processed products accounts for almost 39% of the entire egg market [3]. Three primary parts make up an egg: the shell (90–120 g/kg), white (600 g/kg), and yolk (300–330 g/kg). The components of a whole egg include water (750 g/kg), lipids (primarily concentrated in the yolk, 120 g/kg), highly digestible proteins (mostly dispersed among the egg white and the yolk, 120 g/kg), and carbohydrates, minerals, and vitamins (10 g/kg) [4,5]. Egg yolk (EY) is an efficient dietary ingredient owing to its nutritional (essential fatty acids, phospholipids, minerals, and vitamins) and organoleptic (rich flavor) qualities [6]. The fresh EY components were 50.1% water, followed by 30.6–35% lipids, 15.0–17.0% proteins, 0.6–1% carbs, and 1.7% minerals. The EY protein sector consists of low-density lipoproteins (LDLs, 65%), lipovitellin (16%), livetin (10%), phosvitin (4%), egg yolk riboflavin binding protein (RBP, 0.4%), yolk immunoglobulins (IgY), and others (4.6%) [7,8]. In its natural state and from the standpoint of solubility, EY is a complicated system composed of insoluble granules (ranging in size from 0.3 × 10^−6^ to 2 × 10^−6^ m) suspended in a transparent yellow fluid (plasm) that can be easily separated by centrifugation [9,10]. In contrast to the plasma, which accounts for 78% of the EY and contributes 90% of its lipids, only approximately a quarter (22%) of the yolk is found in the granules, where it contributes approximately 50% of the EY proteins and the remaining 7% of its lipids [9,11].

Egg white EW is commonly utilized as a practical and easily accessible food ingredient throughout processing because of its essential amino acid supply, high bioavailability, and remarkable functions (such as gelling, forming, and emulsifying capabilities) in the culinary and food sectors [12,13]. The majority of EW consists of water (88%), followed by protein (11%), and also presents trace levels of ash, carbs, and lipids (1%). The predominant proteins in EW comprise ovalbumin (54%), ovotransferrin (conalbumin) (12%), ovomucoid (11%), G_2_ globulin (4%), G_3_ globulin (4%), lysozyme (3.5%), and ovomucin (3.5%), ovoinhibitor (1.5%), ovoglycoprotein (1%), thiamin binding protein (1%), ovoflavoprotein (0.8%), ovomacroglobulin (Ovostatin) (0.5%), with only trace quantities of avidin (0.05%) and cystatin (0.05%) [12]. EW proteins possess a wide range of biological properties intimately associated with human health, and their functional activities are widely applicable in the biological, food, and pharmaceutical sectors. For instance, lysozyme, which has excellent antibacterial action, is employed as a food preservative to prevent the growth of microbial pathogens while manufacturing meat products [14,15].

Eggs are a common and potentially serious food allergen, with EW being the primary source of allergenic proteins affecting many children and adults. Especially in children, it often develops during the first year of life. However, many children will outgrow their egg allergy by the time they reach school age [16,17,18]. The prevalence of egg allergy varies widely across different populations and geographical regions, likely due to differences in dietary habits, genetic factors, and environmental exposures. Studies have also shown that egg allergy is prevalent in individuals with other allergic conditions, such as atopic dermatitis, asthma, and allergic rhinitis [19,20,21,22].

In addition, egg proteins are a rich source of bioactive peptides and short chains of amino acids with specific biological activities. These peptides are produced by the enzymatic hydrolysis of egg proteins and have been reported to have various health benefits, including antioxidant, anti-inflammatory, and antimicrobial activities [23,24]. However, the potential allergenicity of these peptides is a concern, as they may trigger allergic reactions in certain individuals. Various factors, such as the protein source, processing conditions, and individual sensitivity, influence the allergenicity of proteins. When ingested, the body’s immune system identifies these proteins as harmful and produces antibodies, releasing histamine and other chemicals that cause an allergic reaction. Egg allergy symptoms can range from mild to severe, including hives, eczema, wheezing, vomiting, and anaphylaxis [25,26,27,28].

While some egg-derived peptides are safe for consumption, others may pose a risk to individuals with egg allergies. For example, a study by Wang et al. (2017) found that a peptide derived from ovotransferrin could bind to IgE antibodies from patients with egg allergies, indicating that the peptide may be allergenic [29]. In contrast, another study by Ma et al. (2020), found that a peptide derived from ovotransferrin could not bind to IgE antibodies, suggesting that the peptide may be less allergenic [30].

The prerequisite for utilizing the biological activity of egg proteins is the extraction of proteins from the egg. Hence, developing effective extraction methods is one of the cornerstones of understanding and studying egg protein structure and activity. In addition, eggs are not only considered foods that can be consumed directly, but EWs and/or EYs are also used as functional and nutritional ingredients in a wide variety of nutraceutical and pharmaceutical products, owing to their numerous biological and functional characteristics [31]. Eggs are therefore processed in different ways before consumption and by producing various products. Their functional characteristics, microbiological quality, shelf life, and protein digestibility are all directly impacted by the processing they undergo [32,33]. On the other hand, several studies have reported the impact of processing and extraction methods on the allergenicity of egg-derived proteins and peptides. For example, heat treatment, commonly used in the food industry, can denature egg proteins and create new allergenic epitopes that can increase the allergenic potential of the protein [34,35]. Similarly, harsh extraction methods such as acid hydrolysis or high-pressure processing can produce new allergenic peptides by breaking down egg proteins into smaller fragments that may be more potent allergens [36,37]. Conversely, some studies have reported that mild processing methods such as spray drying or freeze-drying can reduce the allergenicity of egg proteins by denaturing the proteins or disrupting their structure [38,39,40]. Generally, processing conditions could impact the allergenicity of egg proteins by influencing the linear and/or conformational epitopes of allergens, enabling the production of hypoallergenic egg products [41,42].

This review will cover three crucial aspects associated with egg proteins, which are: (i) the various techniques used for the fractionation of individual egg proteins; (ii) the bioactive characteristics of peptides generated through enzymatic hydrolysis of egg proteins; and (iii) the possible reduction of allergenicity of egg proteins through different processing methods.

## 2. Methods

In order to conduct this review, a comprehensive literature search was performed using several electronic databases, including PubMed, ScienceDirect, Scopus, and Web of Science. The keywords used in the search were “egg”, “allergen”, “allergenicity”, “processing”, “extraction”, “proteins”, “peptides”, “egg-derived peptides”, “functional properties”, “advanced extraction methods”, and related terms. The search was limited to articles published in English between 2003 and 2023. The resulting articles’ titles and abstracts were screened to identify relevant studies, and full-text articles were then reviewed to determine their eligibility for inclusion in the review. Some articles discussed advanced extraction methods for enhancing the functionality of egg-derived peptides while reducing their allergenicity and excluded articles that did not meet these criteria.

Overall, we identified a total of 486 articles through our systematic search of the literature. After screening and reviewing these articles, we selected 95 to include in the review. The selection of articles was based on the relevance of their content and the quality of the research presented.

## 3. Fractionation of Egg Proteins

**Figure 1:**
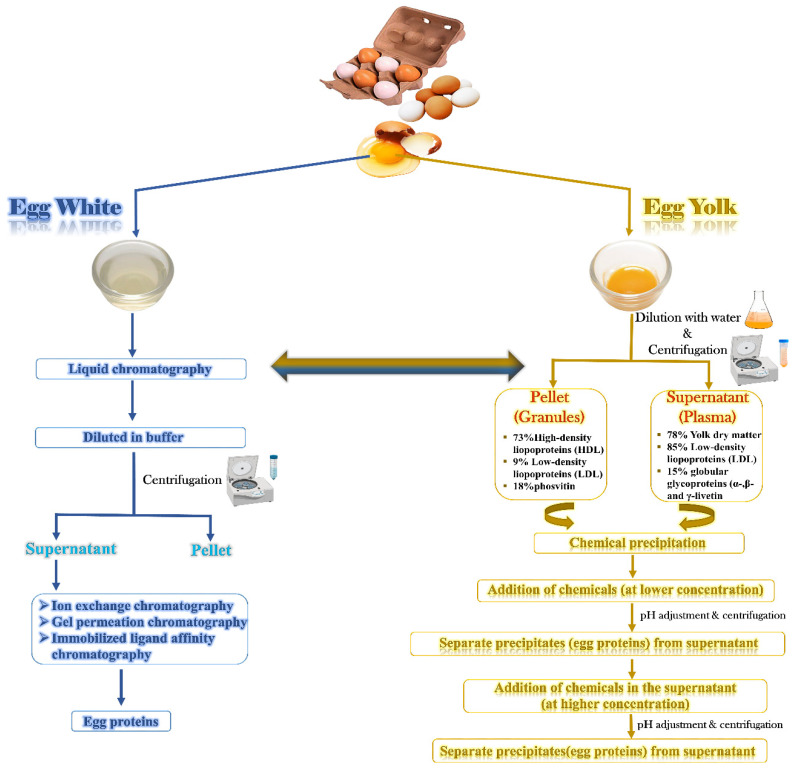
The process of separating egg proteins into fractions using liquid chromatography and chemical precipitation is visually represented.

Egg fractions, the components separated using chemical and physical techniques (as shown in Figure 1), have been studied extensively for their nutritional and industrial properties [43]. Researchers have analyzed their physicochemical properties, such as molecular weight, affinity, surface charges, and acidity, to identify potential applications for these fractions and enhance their value. For example, some egg fractions have been found to have unique functional properties, such as the ability to emulsify or bind ingredients, which make them valuable ingredients in a range of food and industrial products. In addition, egg fractions are also being explored as potential sources of bioactive peptides that could have health benefits, such as reducing blood pressure or improving immune function. Overall, studying egg fractions is an important area of research that can lead to new products and applications and enhance the value of eggs as a commodity [44]. The research on egg fractions is ongoing, and new applications are continually being discovered, making eggs a versatile and valuable commodity.

### 3.1. Fractionation of EW Proteins

**Figure 2:**
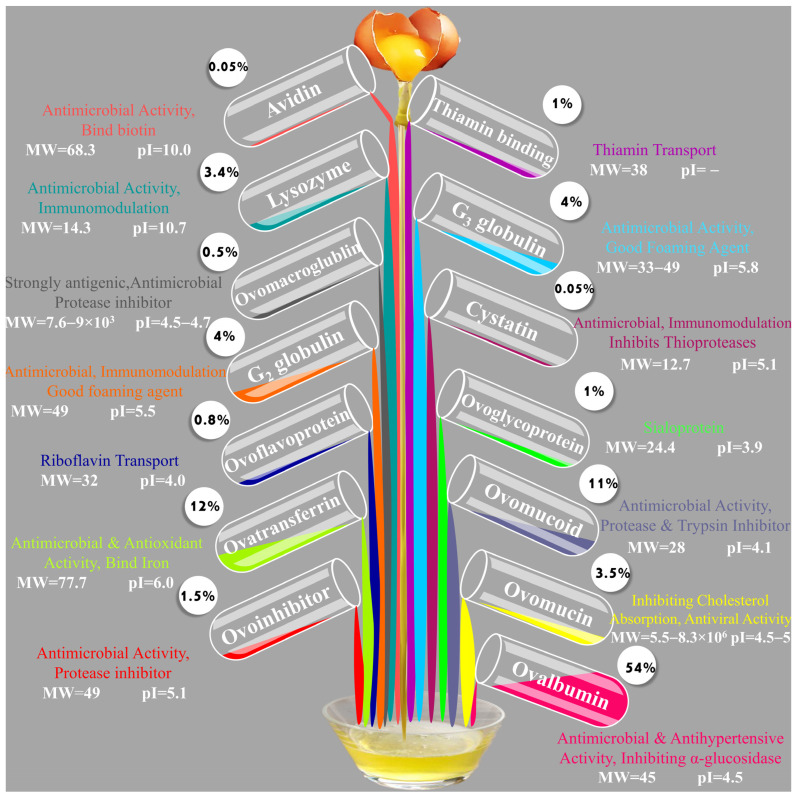
Percentage, Biological activity, molecular weight (MW), and isoelectric point (pI) of egg white proteins.

The most frequently researched EW proteins are ovalbumin (540 g/kg), ovotransferrin (120 g/kg), ovomucoid (110 g/kg), ovomucin (35 g/kg), and lysozyme (35 g/kg). In comparison, minor EW proteins such as ovoinhibitor (15 g/kg), ovoglycoprotein (10 g/kg), ovoflavoprotein (8 g/kg), ovomacroglobulin (5 g/kg), as well as avidin (0.5 g/kg) and cystatin (0.5 g/kg), have received less attention and are studied to a lesser extent [4]. Figure 2 provides the essential characteristics, such as the biological activity, percentage of total protein, molecular weight (MW), and isoelectric point (pI) of both major and minor proteins in EW. Additionally, a concise explanation of their functional properties is presented below.

Ovalbumin, a glycoprotein found in EW, has a molecular weight of 45,000 Da and comprises a chain of 385 amino acids, including 105 titratable residues. It has an N-terminal amino acid of acetylated glycine and a C-terminal amino acid of proline. It is known to have a carbohydrate group attached to its N-terminus [4,45]. OVA comprises three components (A1, A2, and A3) with slightly different electrical properties, with A1 containing 2 mol of phosphoryl groups, A2 containing 1 mol of phosphoryl groups, and A3 containing no phosphoryl groups. Sulfate salting can precipitate OVA at its isoelectric point to obtain needle-like crystals of the protein [12,46]. OVA has excellent functional properties, contributing significantly to the foaming, gelling, and emulsifying properties of EW [47]. OVA is a nutritious protein and has potent antioxidant activity, inhibiting the oxidation of phosphatidylcholine. Covalently binding OVA to polysaccharides increased its antioxidant activity.

Studies have shown that OVA may support immune defense in organisms by providing antimicrobial peptides. These peptides were found to have antibacterial activity against Bacillus subtilis [48,49,50]. Furthermore, OVA is one of the egg allergens, and separating and purifying OVA is crucial for exploring the mechanism and improving egg allergenicity [51,52]. High-purity OVA is often used to investigate the structure and properties of proteins and as an experimental model for allergy [53,54,55]. In addition to its functional and nutritional properties, OVA has also been found to have potential therapeutic applications. Studies have shown that OVA can act as a carrier for drugs and vaccines, leading to enhanced efficacy and delivery to target cells. OVA-based vaccines have been developed for various diseases, including cancer, HIV, and influenza, and have shown promising results in preclinical and clinical trials [56]. OVA has also been investigated for its potential as a biomaterial for tissue engineering due to its biocompatibility, biodegradability, and ability to promote cell adhesion and growth. Furthermore, OVA has been found to have anti-inflammatory and immunomodulatory effects, which may have implications for treating autoimmune and inflammatory diseases. Thus, OVA’s versatile properties make it a valuable protein for basic research and practical applications [57,58].

Ovotransferrin (OVT), or conalbumin, is a glycoprotein of 686 amino acids with a molecular weight of 76,000 Da [9,59]. The amino acid sequence of OVT has been fully characterized and is available in public databases, which can aid in studying its structure and function. It is classified as a monomer and possesses a unique metal-binding ability. It also contains 0.8% mannose and lacks galactose and sialic acid. It accounts for about 12–13% of EW proteins, and each OVT molecule has two ligand centers that can bind to metal ions such as Fe, Cu, or Zn. At pH > 7.0, one molecule of ovotransferrin can easily bind with two iron molecules and subsequently release them into the human body at pH < 4. The iron complex of OVT is stable against protein hydrolysis and thermal denaturation, and it displays a red color when combined with Fe^3+^, with a maximum absorption value near 465 nm. Combining with Cu^2+^ results in a yellow color with a maximum absorption value at 440 nm, while forming a complex with Zn^2+^ increases its thermal denaturation temperature and resistance to proteolytic enzymes and denaturation treatments [60,61]. OVT and lactoferrin share similarities as members of the transferrin family. However, OVT is much more abundant in EW than lactoferrin, making it a promising candidate to compensate for lactoferrin deficiency [62,63]. There are two forms of OVT with distinct physical and chemical characteristics: the apo-form, which lacks iron and is colorless, and the holo-form, which contains a salmon-pink color. The holo-form is more resistant to physical and chemical alterations than the apo-form [62]. The apo- and holo-forms of OVT actively enhance iron absorption without accumulation or gastrointestinal irritation, making it an excellent iron absorption enhancer. Moreover, iron bioavailability from OVT is higher than from other iron sources, such as ferrous sulfate. This makes it a potentially useful iron supplement for individuals with iron-deficiency anemia [64]. OVT can also maintain the integrity of the gastrointestinal barrier, making it ideal for dietary supplementation strategies [65,66]. In addition, OVT has been found to have antimicrobial properties against a wide range of pathogens, including bacteria, fungi, and viruses. This makes it a promising candidate for developing new antimicrobial agents. The main explanation for OVT’s antimicrobial effect is that it binds to the iron needed by iron-demanding microorganisms, such as *Shigella dysenteriae*, and chelates it, thereby inhibiting growth. OVT can also suppress *Salmonella*-induced egg infections by mediating iron chelation-induced iron deficiency. Another theory is that OVT directly interacts with chelating divalent ions on the outer membrane surface of gram-negative bacteria, causing membrane perturbation and inhibiting bacterial growth. The inhibitory effect of OVT is influenced by various factors in the natural environment of EW, including alkalinity, high viscosity, and ionic composition, as well as synergistic effects with other proteins [67,68]. In addition, OVT has some antioxidant activity, but this can be enhanced by adding natural plant extracts such as vitamin C, caffeic acid, and quercetin, or by hydrolyzing OVT to obtain antioxidant peptides. Hydrolysates of OVT, such as those produced by the promod 278P enzyme, have been found to have high ACE inhibitory activity and potential for use in anti-cancer and anti-hypertensive drugs [69,70]. OVT has also been investigated for its potential to prevent or treat various diseases, including osteoporosis, neurodegenerative disorders, and cancer. It has been found to modulate the immune system and reduce inflammation, which may have applications in treating inflammatory bowel disease and other immune-related conditions [71,72].

Lysozyme (LYS) is an egg white protein with a strongly alkaline nature and a molecular weight of 14,400 Da. It is typically found in monomeric form and comprises a single chain of 129 amino acids. It tends to bind to negatively charged proteins, such as ovomucin [73,74]. Its N-terminal amino acid is lysine, and its C-terminal amino acid is leucine. The presence of four disulfide bridges in LYS contributes to its ability to withstand high temperatures, resulting in high thermal stability. Isoelectric point precipitation and cation exchange methods effectively separate LYS from other proteins. In nature, LYS is widespread, with saliva, tears, mucus, human milk, and EW being the main sources. Lysozyme from EW is the most studied due to the easy availability of materials, low cost, and high content [75,76].

LYS is known for its ability to hydrolyze the peptidoglycan of the bacteria’s cell wall and is commonly used as a preservative and bactericide. It is more effective against gram-positive bacteria due to their higher peptidoglycan content. Various modification approaches have been introduced to expand its antimicrobial spectrum. The activity of LYS decreases with heat denaturation, but its antimicrobial effect on certain gram-negative bacteria is enhanced. LYS is widely used in the food industry, including kimchi pickling, sushi, Chinese noodles, and cheese [77,78,79]. In the pharmacological field, LYS has extensive use in wound-healing creams, eye drops, anti-cancer drugs, and as an antiviral agent and immunomodulatory [80,81,82,83]. Recent studies have shown that a non-peptide fragment (HL9) from human LYS has anti-HIV-1 activity at nanomolar concentrations [84,85]. LYS, OVT, and lactoferrin may also effectively treat or prevent COVID-19, acting as antiviral agents and immunomodulators [86].

Ovomucin (OVN) is another important glycoprotein found in egg white, which has both soluble and insoluble components weighing 8300 Da and 220,000–270,000 Da, respectively. OVN contains 330 g/kg of carbohydrates, such as galactose and galactosamine, attached to its structure, and it is distributed throughout the gel-like structure of EW. OVN comprises two subunits. The first is α-ovomucin, consisting of acidic amino acids such as aspartic and glutamic acid with fewer (10–15%) carbohydrate groups. The second subunit, β-ovomucin, is a heterogeneous subunit primarily consisting of serine and threonine, with more (50–65%) attached carbohydrates connected by disulfide bonds [87,88,89]. The glycoprotein known as OVN boasts an impressively dense glycosylation profile, with over 30% of its structure comprising these sugar molecules. Its high molecular weight and viscosity are also noteworthy features. In conjunction with its association with LYS and globulin, this renders OVN an exceptional candidate for generating long-lasting foam. OVN stands out for its superior ability to generate foam compared to bovine serum albumin. This makes it a popular foam stabilizer in food processing applications where optimal foaming capacity is desired [90,91]. Furthermore, research has revealed that OVN exhibits antimicrobial, antiviral, anticancer, and macrophage-stimulating properties. The concentration of OVN is four times higher in the dense EW than in the diluted version. When the pH level reaches around 9, the binding of OVN and LYS results in the dilution of the dense EW [90,92].

Ovomucoid (OVM) is a glycoprotein that is present in egg white. It has a molecular weight of 28,000 Da and contains sialic acid, approximately 4.5% mannose, and roughly 1.5% galactose. The three-dimensional structure of OVM features three disulfide bonds, which means it has nine disulfide bonds and does not contain any free sulfhydryl groups. These disulfide bonds play an important role in the physiological activity of OVM. The protein comprises three regions, I, II, and III, with region II responsible for inhibiting trypsin [93,94].

The inhibitory activity of OVM is related to its binding with trypsin in a 1:1 ratio. The disruption of OVM’s disulfide bond results in the loss of its inhibitory function. However, the activity can be restored upon re-oxidation. It has been found that OVM retains an exceptional level of stability in its trypsin-inhibiting function even after being subjected to heating at a pH of 7 or lower. The trypsin inhibitory function of OVM has important in vitro applications, as it can be utilized to prevent excessive hydrolysis resulting from trypsin addition by incorporating it as an additive. OVM’s ability to inhibit trypsin protects the developing embryo by suppressing bacteria, as the proteases necessary for bacterial growth are inhibited. OVM is also commonly used as an alternative to serum trypsin inhibitors in cell culture to prevent cell loss and death after cell dissociation [95,96]. However, OVM’s allergenicity is a significant area of research. It is well known that OVM is responsible for egg allergies, as it can inhibit trypsin and bind to immunoglobulin E (IgE) even when broken down into smaller peptides. OVM may play a more important role in the pathogenesis of EW allergic reactions than other EW proteins. Several modification experiments, such as covalent bonding, superheated steam treatment, and electrolysis, can effectively reduce the allergenicity of OVM [97,98,99]. Additionally, OVM-derived peptides are biologically active, and using suitable enzymes for OVM hydrolysis can yield active peptides for use in nutritional preparations or food processing. Compared to bovine-derived proteins, egg-derived trypsin inhibitors in food have a higher safety profile [100,101,102,103].

In addition, ovomacroglobulin is a protease inhibitor that is present in a small amount (0.5%) in egg white and is widely used for this purpose [62]. Its molecular weight is the second largest among egg glycoproteins after OVN. Ovomacroglobulin consists of four subunits of equal molecular weight (SDS-PAGE analysis indicates approximately 175 kDa), two of which are linked by disulfide bonds. Various biological functions of ovomacroglobulin have been reported, including antibacterial and anti-inflammatory effects, keratitis treatment, and hemagglutination inhibition [104].

An avidin protein is a glycoprotein found in small amounts (0.05%) in EW. Avidin comprises four identical subunits, each with a molecular weight of 15.6 kDa and consisting of 128 amino acids. This protein has a strong affinity for biotin and can bind to it specifically [105]. Avidin is not heat-stable and can be irreversibly denatured at 70 °C. However, the complex formed between avidin and biotin remains stable at 100 °C and requires a temperature of 120 °C for 15 min to break down. The strong binding between avidin and biotin is widely utilized in various fields, including molecular biology, molecular recognition and labeling, enzyme-linked immunosorbent assays (ELISA), histochemistry, and cytochemistry [106].

### 3.2. Separation Methods of EW Proteins

The exceptional characteristics and diverse functionalities of egg white proteins have made them valuable in the food industry. Various methods (Table 1) have been developed to separate and refine each protein component, showing significant potential. Liquid chromatography techniques such as gel permeation, ion exchange, and immobilized ligand affinity chromatography have effectively fractionated egg white proteins. Among these, ion exchange chromatography is most widely used due to its high production capacity and ease of scaling up for industrial applications. In addition to liquid chromatography, other methods have been used to separate EW proteins, including electrophoresis, precipitation, ultrafiltration, and membrane separation [4,12].

#### 3.2.1. Chromatography

Chromatography is a widely used technique for separating complex mixtures of substances. The basic principle involves selectively distributing different components between the stationary and mobile phases, leading to the elution of the mixture in the stationary phase. Different components move along the stationary phase at different speeds, resulting in separation. Chromatography has several advantages, including high separation efficiency, minimal raw material usage, high sensitivity, fast analysis speed, and ease of operation. Chromatography is frequently employed in separating egg white proteins and can be categorized into ion exchange, gel filtration, affinity, and adsorption chromatography based on the separation mechanism. Each of these methods is explained and illustrated below [107].

##### Ion Exchange Chromatography

Ion exchange chromatography is a technique that separates molecules based on their charge. It can adjust the charge distribution by altering the pH and ionic strength in the mobile phase. The choice between cation or anion exchange chromatography depends on the target protein, as some proteins are more sensitive to changes in pH [108]. For EW protein analysis, both cation and anion exchange chromatography have been used, although anion exchange chromatography is more commonly used for EW protein separation. This method separates components by their selectivity coefficients using an ion exchanger as the stationary phase, with a pre-loaded column or suitable ion exchange medium selected during the purification of EW proteins. Cation exchange chromatography is frequently employed to separate and purify LYS from other major egg white proteins due to its abundance of essential amino acids, resulting in a positive charge. In contrast, most other proteins have a negative charge [109,110].

LYS, OVT, and OVA were fractionated by Vachier et al. using anion-exchange chromatography with a Q-Sepharose fast-flow column. They were able to achieve protein recovery (>62%) and purity (>75%) [4,111]. Awadé et al. utilized gel-permeation chromatography to isolate OVN and lysozyme from native egg white diluted in a 0.05 mol L–1 tris(hydroxymethyl) aminomethane hydrochloride buffer (pH 9) containing 0.4 mol L–1 sodium chloride (NaCl). They obtained high purity of ovomucin (80%) and lysozyme (>99%) by exploiting the differences in molecular size and hydrodynamic volume in the solvent [4,112]. Compared to ion-exchange chromatography, gel-permeation chromatography is less suitable at a process level as it requires the removal of lipids from egg protein sources prior to loading on the column. This is not necessary for ion exchange chromatography [4,113]. However, gel-permeation chromatography does not affect protein structure, and initial activity is maintained after separation.

A new light-sensitive cation exchanger, PNBCC, was created by randomly synthesizing acrylic acid, copolymerizing chlorophyllin sodium copper salt, n-butyl acrylate, and N-isopropyl acrylamide [114]. This exchanger was employed to purify LYS, resulting in a 16-fold increase in LYS activity compared to the original EW protein solution. Magnetic chitosan (MCHT) beads were created through phase inversion and were then grafted with poly(glycidyl methacrylate) (p(GMA)) via surface-initiated atom transfer radical polymerization (SI-ATRP) [115]. In the presence of sodium sulfite, the grafted polymers’ epoxy groups were altered into a strong cation exchange group, such as sulfonate groups. The magnetic cation exchange beads effectively purified LYS [116]. A cation exchange matrix with zwitterionic and multimodal properties was synthesized using a reaction sequence in which sulfanilic acid was coupled to chitosan-based support. This chromatography matrix was cheap, recyclable, and easy to use. Additionally, two diatom shells, AQ1 and NP, were utilized as cation exchange matrices, and their LYS purification effects were assessed. The findings demonstrated the feasibility of ion exchange chromatography for LYS separation. At the same time, the technique was rarely used alone to purify other EW proteins, as will be explained in subsequent sections [117].

##### Affinity Chromatography

Affinity chromatography utilizes the affinity between a substance to be separated and its specific ligand. This method offers the advantage of one-step purification, which is less time-consuming and complex than other methods. Bioligands are more specific, but also more expensive and difficult to maintain activity during the separation process. Conversely, dye ligands are cheaper and more readily available, and they can be immobilized on polymeric substrates by covalent bonding [118,119]. Immobilized-ligand-affinity chromatography is a technique that selectively isolates individual proteins from samples by forming a complex between the protein of interest and a ligand attached to a resin. Al-Mashikhi and Nakai successfully used this method to extract ovotransferrin from egg white proteins using a Cu-Sepharose 6B column, resulting in a high yield of recovery (95%) and protein purity (>94%) with just one step. However, using extreme elution conditions to break the strong interaction between the proteins and the columns may cause protein denaturation. Moreover, immobilized-ligand-affinity chromatography costs are relatively high, making it less feasible for large-scale processes [120].

New methods have been developed to improve the separation of LYS, such as a functionalized graphene-based composite and magnetic chitosan microspheres modified with a reactive red 120 affinity dye ligand. These approaches can recover up to 90% of LYS and are often used to concentrate and refine trace amounts of LYS. While there are more reports on purification by affinity chromatography for LYS, there are fewer reports related to other EW proteins. The key challenge for affinity chromatography in protein separation studies is achieving repeatable stability of affinity-packed columns to reduce costs and improve purification efficiency [121].

##### Adsorption Chromatography

Adsorption chromatography is a separation technique that utilizes a solid stationary phase. It relies on the varying adsorption properties of the solid adsorbent on the substance to be separated. Adsorption mainly involves intermolecular forces such as hydrophobic forces, aromatic ring interactions, and hydrogen bonding. When the adsorbate is exposed to specific conditions, it can be removed from the adsorbent surface, a process known as desorption. The separation process in adsorption chromatography involves a series of repeated adsorption and desorption cycles [122].

A column made of a hydrophobic poly (hydroxyethyl-methacrylate-N-methacryloyl-L-phenylalanine) (PHEMAPA) bead embedded in a poly (hydroxyethyl-methacrylate) (PHEMA)-based cryogel was synthesized using suspension polymerization. This column is ideal for purifying LYS through hydrophobic interactions [123]. A small packed bed using 40 mM carbonate buffer (pH 12) and 0.5 M NaCl as an eluent was used to elute LYS, leading to better LYS recovery from high viscosity EW [124]. Similarly, an affinity cryogel was created through covalent immobilization, which increased the pore size and mechanical strength of the cryogel, resulting in a high LYS adsorption capacity [125]. STREAMLINE SP and SP-XL high-density adsorbents were utilized as adsorbent carriers to optimize the adsorption conditions for LYS. These two adsorbents were ideal for directly recovering LYS from crude EW solutions. While adsorption chromatography is highly efficient for purifying LYS, it is less commonly used for purifying other proteins [126].

##### Gel Filtration Chromatography

Gel filtration chromatography, also known as size exclusion chromatography or molecular sieve chromatography, is a technique used to separate molecules based on their size [127]. The mobile phase used in this method is either water or a buffer solution, and it takes advantage of the molecular sieve effect of the network structure gel to separate molecules according to their size. Unlike other techniques, this method does not require any precipitants, such as organic solvents that can cause protein denaturation, during the process. In general, it is assumed that the separated proteins have the same symmetry and that the protein with a higher molecular weight is eluted first, while the protein with a lower molecular weight is eluted later [128].

Gel filtration chromatography is particularly useful for separating proteins with large differences in molecular weight from other proteins, such as OVN and ovomacroglobulin, in egg white. When separating OVN, a pretreatment process is necessary to reduce the viscosity and prevent column clogging. Wang et al. (2018) used a two-step salting-out process and Sephacryl S-300 HR gel filtration chromatography to purify OVN with high antiviral activity. This method overcomes the difficulties of separating OVN due to its high molecular weight, viscosity, and susceptibility to degradation [129]. Gel filtration chromatography also provides a method for separating ovomacroglobulin. It involves obtaining a precipitate enriched with ovomacroglobulin by PEG precipitation and then purifying it by two-step chromatography using a Q Sepharose Fast Flow anion-exchange column and a Sephacryl S-200 HR gel filtration column [106]. A one-step chromatographic method using a Sephacryl S-200 gel column can achieve 97.0 ± 0.3% purity of ovomacroglobulin [130]. This method simplifies the complex chromatographic process, and the entire purification process can be completed within one day.

Gel filtration chromatography has advantages in that it provides better separation, allows for entire separation in one column volume, and does not cause protein denaturation. However, it requires longer columns, resulting in slower flow rates, longer elution times, higher column pressure, higher column material requirements, and higher packing costs. Therefore, gel filtration chromatography is often used with other methods to reduce separation time and prepare high-purity proteins [131].

Besides liquid chromatography, adding chemicals such as ammonium sulfate, ethanol, and calcium chloride with or without physical procedures such as electrophoresis and ultrafiltration is another important approach for separating EW proteins. For example, Omana and Wu investigated using different concentrations of calcium chloride and potassium chloride, combined with isoelectric precipitation, to extract OVN. Salts can alter the ionic species and concentrations, affecting electrostatic interactions among proteins and protein solvents, ultimately determining protein solubility. The authors found that the highest purity (97.3%) of OVN could be produced using a two-step method, in which 0.05 mol L^−1^ calcium chloride was first used to precipitate EW proteins, followed by the addition of 0.5 mol L^−1^ calcium chloride extraction. However, using potassium chloride resulted in high impurities with OVT and low sample handling capacity [132]. OVT and OVM were fractionated from EW by treating them with a high ethanol concentration and acidic salt precipitation based on their solubilities in those solutions. A 430 g/kg ethanol solution was first used to treat egg whites, followed by centrifugation. The supernatant was then precipitated using 610 g/kg ethanol to obtain ovotransferrin (purity > 88%) or 25 g/kg ammonium sulfate and 25 g/kg citric acid to obtain ovomucoid (purity > 89%). The production yields of OVT and OVM were >92% and >96%, respectively. This approach can easily be scaled up with a high production yield [133].

#### 3.2.2. Precipitation

Proteins can be separated using precipitation techniques that rely on solubility or isoelectric point differences. Salting-out, organic solvent precipitation, and isoelectric point precipitation are the most commonly used methods for precipitating egg proteins. Salting-out involves the separation of proteins in highly concentrated salt solutions, often followed by a desalting process to obtain pure proteins. Neutral salts are typically used to purify egg white proteins to prevent protein denaturation. Organic solvent precipitation reduces protein solubility in water by using solvents that are miscible with water, but a high concentration of organic solvents can damage proteins and pose safety risks. Isoelectric point precipitation separates proteins based on their minimum solubility at their isoelectric points, but the method has limited application due to the similar isoelectric points of most EW proteins [134]. Thus, it is often used in conjunction with other techniques. Although precipitation methods are simple and quick, they are challenging to scale up for industrial use. The precipitation of egg white proteins is typically done using ammonium sulfate due to its high solubility, low-temperature coefficient, and low denaturation of proteins. In one study, OVT was precipitated with ammonium sulfate and citric acid, followed by desalting through ultrafiltration, resulting in a yield of over 83% and a purity of over 85%. Since no organic solvent was used, the OVT remained active and could be used for subsequent applications [62]. Another commonly used precipitant is polyethylene glycol. A study reported using PEG combined with isoelectric point precipitation to achieve a purity of 95.1% when separating OVA from EW proteins, processing a kilogram of egg white protein solution in 2–3 h. However, it should be noted that OVA is easily denatured and coagulated by exposure to new surfaces, resulting in a partial loss of activity. The precipitation method is commonly used with membrane separation technology to remove salt or organic solvent. Using it alone has a poor effect, and adding precipitant may cause protein denaturation [135].

#### 3.2.3. Membrane Separation Technology

The use of membrane separation technology for separating EW proteins has been documented in various sources. The most commonly used technique is ultrafiltration, which involves the selective filtering of different solute molecules using a specialized membrane. This method separates large molecules from smaller ones, allowing for the extraction of the desired proteins. Dialysis is another type of membrane separation technique, but it is more suitable for desalting or concentrating smaller volumes of sample solutions and has a longer operating time. However, direct membrane separation technology may not be effective for proteins with similar molecular weights. As a result, it is frequently combined with other techniques, particularly for sample concentration or desalting and devolution after adding a precipitant. For example, in one study, Amberlite FPC 3500 ion exchange resin and precipitation were used to sequentially remove OVT, OVN, LYS, and OVM to obtain an OVA-rich precipitate. This precipitate was then further processed by ultrafiltration to concentrate and desalt it. Finally, ovoinhibitor was obtained through freeze-drying, with a purity of 90.8% and a yield of 70.0%. This method kept the inhibitory activity of ovoinhibitor on various proteases, such as elastase, trypsin, alpha-chymotrypsin, and subtilisin. Overall, membrane separation techniques are generally used as an auxiliary method in the co-purification of multiple proteins, as the direct application may yield a different outcome due to the similar molecular weight of certain proteins [136].

#### 3.2.4. Membrane Chromatography

Membrane chromatography is a technique that combines the principles of membrane separation and chromatography. This method has been identified as a potential alternative to traditional packed-bed chromatography due to its reduced hardware requirements, ease of operation, and shorter processing time [137]. Polyacrylonitrile nanofiber membranes were fabricated to purify LYS using the electrostatic spinning technique, resulting in a 90% capture efficiency and a 47-fold purification factor in a one-step reaction [138]. Similarly, LYS was purified using polyacrylonitrile nanofiber membranes functionalized with tris (hydroxymethyl) aminomethane (P-Tris) as affinity chromatography, which resulted in 93.28% recovery [139]. However, traditional membrane adsorption techniques are limited in several ways, and high-density functional group modification can improve their adsorption capacity. Accordingly, a novel high-capacity tetrazolium-functionalized weak cation exchange membrane was prepared and showed a better binding capacity for LYS and OVT than previously reported cation exchange membranes. Furthermore, Madadkar et al. (2019) compared a laterally-fed membrane chromatography (LFMC) device with a strong cation exchange membrane with an equivalent conventional resin-filled column. They found that the LFMC device had a significantly higher theoretical column number. This device could separate a ternary model of a protein mixture consisting of OVA, OVT, and LYS with significantly better resolution than conventional columns. In conclusion, membrane chromatography has advantages not found in individual membrane separation techniques or chromatography, and it has good prospects in protein purification [140].

#### 3.2.5. Electrophoresis

Electrophoresis is a laboratory method that exploits the differential movement of charged molecules in an electrical field to isolate the desired protein from a mixture of impurities. It relies on the fact that proteins carry a net charge and will migrate toward the electrode of the opposite charge, thereby allowing the separation of proteins based on their electrical properties. Two main electrophoresis methods used for separating EW proteins include isoelectric focusing and free-flow electrophoresis. Shimazaki et al. (2018) employed non-denaturing two-dimensional electrophoresis (2DE) to separate and transfer LYS-binding proteins to membranes, finding that the LYS-OVT complex had lysis activity against both Bacillus subtilis and Escherichia coli [141]. Non-denatured gel isoelectric focusing also effectively separated natural proteins that retained biological activity [142]. Isoelectric focusing was found to have high resolution and be capable of separating proteins whose isoelectric points differ by 0.01–0.02 pH units. Free-flow electrophoresis combined with gel filtration chromatography successfully isolated low-abundance LYS [143]. A special polyacrylamide-co-acrylic acid gel electrophoresis combined with mass spectrometry was used to identify prepared LYS. Gel electrophoresis is commonly used to identify and analyze proteins in samples to determine their content and purity. As only small amounts of proteins are usually prepared, gel electrophoresis is used in combination with other separation techniques. Additionally, free-flow electrophoresis and isoelectric focusing can be combined to form free-flow isoelectric focusing, which provides the advantages of mild separation conditions, high recovery, and high resolution. Wang et al. (2019) constructed a homemade carrier ampholyte-free-flow isoelectric focusing system that used directed migration of H^+^ and OH^−^ provided by electrode solutions for the separation of OVM, OVA, and OVT. This study effectively attempted to solve the problem of the high cost of carrier amphoteric electrolytes. Electrophoresis is generally used to extract small amounts of proteins, although it can be employed to separate EW proteins [144].

#### 3.2.6. Aqueous Two-Phase Systems

An aqueous two-phase system is an innovative approach to protein recovery on a large scale. Recent advancements in this method have opened up new possibilities for industrial protein production and downstream processing. Polyethylene glycol is highly hydrophilic and can induce protein aggregation and precipitation, making it ideal for use in aqueous two-phase systems. The PEG/phosphate system has been used to purify avidin from EW, achieving a purification factor of 5.7 and a yield of 92% in the most efficient small-scale aqueous two-phase system [145]. In another study, OVA was purified using an aqueous two-phase system consisting of PEGs of different molecular weights and an aqueous potassium citrate/citric acid buffer [146]. The method achieved sustainable OVA recovery at a low cost in a single step and can be easily scaled up for industrial use. Additionally, ionic liquids have been employed to purify EW proteins in aqueous two-phase systems. Ionic liquids are stable, tasteless, and pollution-free, and have rapidly gained popularity in protein purification due to their environmentally friendly nature. A novel acrylonitrile-butadiene-styrene copolymer consisting of a tetraalkylammonium-based ionic liquid and a potassium phosphate solution was developed to recover 99% of LYS from the ionic liquid-rich phase. This study has expanded the scope of ionic liquid applications and introduced new options for solvents used in protein precipitation [147]. However, despite its potential, the aqueous two-phase system has yet to be widely used for commercial applications. Further research is needed to fully explore its potential for protein purification.

#### 3.2.7. Molecular Imprinting Technology

Molecular imprinting, known as molecular template technology, employs template molecules to create an imprinted cavity within a polymer structure. This results in the formation of shape recognition and chemical selectivity for the target molecule, which has proved useful in separating EW proteins [148]. This technology has developed various techniques, including a hollow embossed silica polymer with covalently bonded modified proteins on its surface and a novel membrane containing LYS recognition sites prepared by surface-initiated atom transfer radical polymerization [149,150]. Wang et al. (2014) also developed an LYS molecularly imprinted polymer through embedding, which had twice the adsorption capacity of the non-molecularly imprinted polymer [151]. Magnetic molecularly imprinted polymers have been shown to be ideal for the specific separation of biomolecules. For instance, a core-shell nanocomposite was developed for the specific recognition of LYS, which was easily prepared, had good selectivity and high binding ability, and was stable enough to be reused without degradation in performance [152]. Similarly, Xu et al. (2018) developed a magnetized molecularly imprinted polymer that could be used to specifically identify LYS, which showed that the polymer could be repeatedly adsorbed four times without causing a significant decrease in adsorption capacity. Molecular imprinting technology has a promising future in purification due to its specific selectivity for target molecules. Although currently, molecular imprinting technology is most frequently used in isolating LYS, for target proteins with known structures and properties, molecular imprinting can be considered, which may be able to achieve high purity [153]. Although the implementation of these methods has yet to mature and the reported effects of their application are less detailed, these studies indicate the potential of these novel methods in the separation and purification of EW proteins.

### 3.3. Separation of EY Proteins

**Figure 3:**
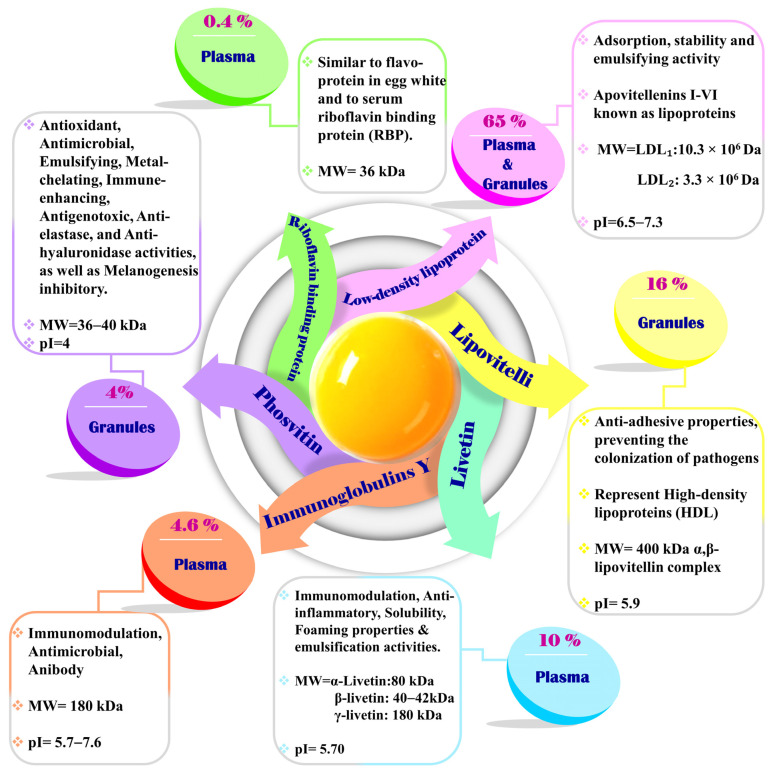
Percentage, location, Biological activity, molecular weight (MW), and isoelectric point (pI) of major egg yolk proteins.

The proteins found in egg yolk (Figure 3) are distributed in two main forms: granules and plasma. The granules contain lipo vitelline (700 g/kg), phosvitine (160 g/kg), and low-density lipoproteins (120 g/kg), while the plasma contains low-density lipoproteins (850 g/kg) and livetin (150 g/kg) [9].

Separating these two forms can be easily achieved by diluting and centrifuging the egg yolk. McBee and Coterill’s method, which involves diluting EY in a 0.17 mol L^−1^ NaCl solution and centrifuging it at 10,000× *g* for 15 min, is still the most commonly used technique [154]. Strixner and Kulozik have recently proposed an alternative method that involves diluting the egg yolk in a 0.15 mol L^−1^ NaCl solution at a 1:2 ratio, stirring the mixture for 1 h at 10 °C, and then centrifuging it at 10,000× *g* for 45 min [10]. This method has shown promising results for industrial-scale fractionation of EY. Table 2 provides an overview of various methods for isolating egg yolk proteins.

EY contains liposoluble glycoproteins called lipovitellins, which are α-lipovitellin and β-lipovitellin. These proteins precipitate at different pH levels, with α-lipovitellin precipitating at pH 7.5–7.8 and β-lipovitellin precipitating at pH 6.5–7.0 [155]. These proteins play important roles in the metabolism and storage of lipids and the transport of nutrients to the developing embryo. Recent studies have suggested that lipovitellins may have significant health benefits. In particular, lipovitellins have been shown to potentially prevent atherosclerosis, a condition characterized by the buildup of cholesterol and other substances on the walls of blood vessels. Atherosclerosis is a major risk factor for heart disease and stroke, the leading causes of death worldwide. The mechanism by which lipovitellins may prevent atherosclerosis is not yet fully understood. However, it is believed to be related to their ability to bind to cholesterol and prevent its accumulation on the walls of blood vessels. This hypothesis has been supported by studies in which lipovitellins were found to reduce cholesterol accumulation in cultured cells and animal models of atherosclerosis [156,157].

Despite their potential health benefits, the production of lipovitellins currently needs to be improved due to several challenges. In particular, the current production methods have a low yield, making it difficult to produce lipovitellins on a large scale. A study by Luo et al. demonstrated that the yield of lipovitellins using the current production method is only 21%, limiting their potential for use in food and pharmaceutical applications. The study involved separating granules from EY through NaCl washing and centrifugation and then dissolving the granules in a NaCl solution. Ammonium sulfate was added to the solution to precipitate the lipovitellins, which were then separated through centrifugation at 11,739× *g* for 30 min [158].

The literature suggests that lipovitellins can only be produced on a small scale due to the lack of a viable production method [158]. To overcome this challenge, researchers are exploring new methods for the production of lipovitellins. One promising approach involves using enzymes to break down the complex lipovitellin proteins into smaller, more easily extracted peptides. Another approach involves using genetically modified chickens that produce high levels of lipovitellin in their eggs.

Phosvitin, a protein found in egg yolk, exhibits unique properties that offer a wide range of potential applications in the food industry and beyond. It comprises two subtypes, α-phosvitin and β-phosvitin, aggregates of subunits with molecular weights of 35,000–40,000 Da and 45,000 Da, respectively [159,160,161]. Apart from its high phosphorylation, phosvitin has remarkable emulsification, antioxidant, antibacterial, and metal chelation abilities, making it a valuable ingredient in the development of functional foods [162,163].

Phosvitin’s significant application as an emulsifier can create stable oil and water emulsions, which are particularly beneficial for producing dressings, sauces, and baked goods [164]. Moreover, it can be an antioxidant, protecting food products from oxidative damage and prolonging their shelf life. It exhibits antibacterial properties, making it an ideal component in antimicrobial packaging materials for mitigating the growth of harmful bacteria and preserving food [165,166,167].

Phosvitin’s ability to chelate metal ions makes it beneficial in various applications, such as water treatment, where it can remove heavy metals from contaminated water sources [168]. Phosvitin has also been studied as a drug delivery agent owing to its ability to bind to metal ions and biocompatibility [169]. Furthermore, research suggests that phosvitin may exhibit anti-inflammatory and immunomodulatory effects, making it a valuable ingredient in functional foods and dietary supplements [69,170].

Various techniques have been employed to extract and purify phosvitin. Ren and Wu (2014) have extracted phosvitin from EY granules using a 100 g/kg NaCl solution at pH 7.25 and adjusted the pH to 3.5 to remove impurities. They discovered that decreasing the pH from 8.0 to 5.5 could enhance the purity of phosvitin from 545 g/kg to 637 g/kg, but the maximum recovery rate of 82.7% was achieved at pH 8.0. Anion-exchange chromatography was employed to purify the crude phosvitin further, resulting in a purity of 97.1% with a 42.0% recovery rate, a method that can be employed in the industry [171]. Ko et al. (2011) developed partially purified phosvitin by first separating yolk granules containing phosvitin from the yolk by adjusting the pH to 4.0–8.0 and centrifugation. They subsequently removed lipids and phospholipids using 85% ethanol, generating lipid-free granules homogenized with ammonium sulfate or NaCl at pH 4.0 to produce phosvitin with the highest production yield. Finally, the salt was removed by ultrafiltration to achieve phosvitin with a purity of 85% and recovery rates of 72% (by ammonium sulfate) and 97% (by NaCl) [172]. The techniques employed by Ren and Wu (2014) and Ko et al. (2011) provide a basis for the development of improved methods for the extraction and purification of phosvitin, which can contribute to the production of value-added food products [171,172].

Low-density lipoprotein (LDL) is a round molecule that can be found in EY plasma. It contains a core of triglycerides and cholesterol esters, surrounded by a film of proteins and phospholipids. On average, LDLs consist of 830–890 g/kg of lipids, with triglycerides making up 690 g/kg, phospholipids 260 g/kg, and cholesterol 50 g/kg, as well as 110–170 g/kg of proteins. Due to their low density of 0.98 kg/m^3^, LDLs are relatively stable in water-based solutions. In one study, LDLs were extracted from EY plasma by adding 400 g/kg of ammonium sulfate to precipitate livetins and dialyzing the supernatant for 6 h to remove the salt. This resulted in LDLs with a purity of 97% and a yield of 67%. In another study, gel-permeation chromatography was used to isolate LDLs from EY plasma by diluting it with a 2 mol/L NaCl solution and subjecting the mixture to chromatography with an Ultrogel AcA 34 column, followed by elution with a 1 mol/L NaCl solution [155,173].

LDL is commonly known as “bad cholesterol” due to its association with cardiovascular diseases. The LDL molecule found in egg yolk plasma is structurally similar to human LDL and can be used as a model for studying its behavior. In addition, studies have shown that EY LDLs have antioxidant properties, which could have potential health benefits. LDLs have been found to have potential applications in drug delivery and as biomaterials due to their unique structural properties. They have been shown to have a high binding affinity for certain drugs and can be used as carriers for targeted drug delivery. Their biocompatibility and ability to self-assemble into nanoparticles make them promising candidates for various biomedical applications [6,174,175]. In addition to the abovementioned methods, other techniques have been employed to isolate LDLs from EY plasma, such as ultracentrifugation and affinity chromatography. Ultracentrifugation involves high-speed centrifugation to separate particles based on density, while affinity chromatography utilizes specific binding interactions between molecules to separate them from a complex mixture. However, different factors, such as feed composition and breed, can affect the composition and properties of LDLs in EYs. For instance, feeding hens a diet high in omega-3 fatty acids can increase the content of these beneficial fatty acids in egg yolk LDLs [176].

Livetins, a group of globular glycoproteins that exist in α, β, and γ-forms, have a wider distribution than previously thought, as they have also been detected in EY and eggshell membranes in addition to their presence in EY plasma [155]. Among them, γ-livetin is primarily composed of immunoglobulin Y (IgY), which makes it an attractive alternative to mammalian IgG antibodies [177]. Liquid chromatography is a widely employed technique for separating and extracting livetins from EY plasma. However, other techniques, such as ultrafiltration, precipitation, and ion exchange chromatography, have also been utilized to isolate and purify livetins [4]. Researchers have used gel-permeation chromatography with a Sephacryl S-200 column, eluting with a 1.5 mol/L NaCl solution at pH 7.0, to fractionate and recover livetins with a 98% yield. In another study, researchers isolated livetin using gel-permeation chromatography with 4 mol/L NaCl elution, separating it into γ-livetin, α-livetin, and β-livetin. Gel-permeation chromatography has several advantages over other methods for the isolation of livetins from EY plasma, including its simplicity, speed, and high recovery rates [178]. Enzymatic modification of livetins can enhance their functional properties, such as their solubility or stability.

Livetins have gained attention recently due to their potential use in various fields such as food, pharmaceuticals, and biotechnology. Aside from their role in the immune system, they have been found to possess antioxidant, anti-inflammatory, and antiviral properties, making them promising candidates for drug development [179]. The use of livetins as an alternative to mammalian antibodies has gained interest due to ethical concerns surrounding the use of animals in antibody production. Livetins have been successfully used in diagnostic assays for detecting diseases such as avian influenza and salmonella [180]. Livetins can be used as a natural emulsifier in food applications, replacing synthetic emulsifiers that may have negative health effects. In addition to their potential therapeutic uses, livetins have been used in cosmetic formulations due to their skin-moisturizing and wrinkle-reducing properties [181].

Various methods have been developed to extract EY protein isolates to expand the application of EY. In the past, organic solvent washing was used to extract EY protein, often accompanied by phospholipid recovery. However, the use of organic solvents is only sometimes desirable due to the risk of leaving behind organic solvent residues and their incompatibility with food applications. Ethanol is commonly used to separate EY proteins and phospholipids because it has a higher affinity for phosphatidylcholine and phosphatidylethanolamines. For instance, researchers have employed a combination of acetone and ethanol to extract phospholipids (with 800–850 g/kg of phosphatidylcholine and 100–150 g/kg of phosphatidylethanolamines) from fresh egg yolks, followed by filter press separation. Heating methods have also been explored to isolate EY proteins because they are more suitable for food applications and do not leave behind organic solvent residues. However, protein denaturation is a major issue that limits the use of these methods in product development [182,183].

Consequently, alternative techniques to fractionate EY proteins, such as membrane filtration, have been investigated. This method yields high protein yields with minimal protein denaturation. Other methods, including precipitation, ultrafiltration, and ion exchange chromatography, have been used to isolate EY proteins with varying levels of success [184].

Protein isolates with high concentrations of IgY were obtained from EY using precipitation techniques with either ammonium sulfate or sodium sulfate. However, these methods increased the salt concentration in the final products and required multiple operational cycles, leading to higher production costs. An alternative method involving water, ethanol, and hexane was also used, in which water-soluble EY proteins were separated using centrifugation. After separating non-soluble fractions, hexane and ethanol were applied to the mixture, and ultrafiltration was used to isolate the non-soluble EY proteins. However, using organic solvents in this method could lead to protein denaturation, so operating at low temperatures (−5 to 0 °C) is important to minimize this effect [185,186].

Proteins from defatted EY fractions were separated by gel-permeation chromatography based on their molecular size. However, this method is not suitable for industrial applications because the gels used in this process are delicate and can interact with proteins. As a result, ion exchange chromatography has become a more popular large-scale method to separate charged proteins from EY. Castellani et al. (2003) conducted a study in which they employed a two-stage technique involving magnesium sulfate-induced salt precipitation and ion-exchange chromatography to isolate EY proteins mainly concentrated in phosvitin. It is noteworthy that this technique did not involve the use of any organic solvents [187].

Membrane filtration is a cost-effective and easily adaptable method to extract proteins compared to chromatographic methods. The success of protein fractionation depends on factors such as the surface properties of the membranes, pH, and salt concentration. Ultrafiltration is commonly used to remove enzymes, while nanofiltration with negatively charged membranes effectively removes salts from protein fractions [188]. A typical process for membrane filtration includes dilution, filtration, hydrophobic filter delipidation, and diafiltration for desalting and protein concentration. For example, researchers could desalt EY proteins rich in phosvitin using polyethersulfone membranes with molecular weight cut-offs of 10,000 and 30,000 Da [4,188].

## 4. Allergenicity from Egg Proteins

Allergies result from a hypersensitive immune system’s reaction to environmental substances that are generally considered harmless. These substances, termed allergens, encompass a wide range of molecules, including proteins found in various food items, such as cow’s milk, eggs, nuts, fish, crustaceans, shellfish, wheat, soy, and sesame, as well as house dust mites, pet dander, and pollens [189,190]. Upon exposure to these allergens, susceptible individuals undergo an intricate immune response. This response involves the cross-linking of the mast and basophil-bound IgE, which causes the release of allergy mediators and the subsequent activation of immune cells such as T-cells, basophils, and eosinophils. Consequently, this cascade triggers the pathogenesis of allergy symptoms [191,192]. Allergic reactions associated with IgE are categorized as Type 1 hypersensitivity reactions due to their rapid onset and resulting inflammatory immune response upon exposure to allergens [193,194]. Type 1 hypersensitivity reactions encompass a wide range of allergic conditions, including but not limited to anaphylaxis, asthma, eczema, drug allergies, insect allergies, and food allergies [195,196,197,198]. These types of allergies are prevalent worldwide, affecting up to 30% of the global population [199].

Currently, food allergies are the most prevalent type of allergy worldwide. While specific and comprehensive data on the global prevalence of food allergies is limited, the World Allergy Organization (WAO) has estimated, based on data from 89 countries, that over 250 billion individuals throughout the globe are already affected by allergy diseases. This prevalence is expected to increase to 4 billion individuals by 2050 [200,201,202,203]. Food is a crucial source of health and nutrition, yet it is still a mystery why some food items can induce an allergic response in the body, which is the primary focus of research on food allergies. Despite considerable advancements in identifying the underlying mechanisms of allergic reactions, the particular protein components responsible for the allergenicity of foods remain unknown. Eggs are the second most prevalent of the “big nine” dietary allergens (cow’s milk, crustaceans, cereal grains, fish, peanuts, tree nuts, soybeans, and sesame) to cause food allergies worldwide, particularly in infants and young children. Up to 9% of babies are allergic to eggs, while other major food allergies involving peanuts account for 3% and sesame for 0.8% [204,205,206]. Egg allergies develop when the immune system has a hyperactive response to the proteins in the EW and EY [207]. The allergenic potential of EW proteins is greater than that of EY proteins. In 2019, Dang et al. conducted a study that revealed that most infants with egg allergies were sensitized to allergens found in the EW rather than the EY [208]. This finding underscores the importance of investigating all major egg allergens, not just those in the EW. Unfortunately, the current approach to managing egg allergies is strict avoidance, which is difficult because eggs are present in many processed foods and medicines, such as vaccines [209,210]. Additionally, avoiding eggs can lead to nutritional deficiencies since they are a valuable source of essential vitamins, proteins, and fatty acids [211]. On the other hand, research has shown that children who can tolerate cooked eggs may not have a long-lasting allergy, with 80% of children with a raw egg allergy being able to tolerate cooked eggs [212,213,214]. This highlights the need for accurate diagnosis, prognosis, and differentiation between egg-allergic, egg-tolerant, and egg-sensitized individuals, which is crucial for effectively managing egg allergies.

There are various strategies available to increase egg consumption, despite concerns raised by medical professionals regarding potential allergies. One promising approach is the separation of specific egg proteins, followed by the production of bioactive peptides using enzymatic hydrolysis. On the other hand, as bioactive peptides from egg proteins are becoming increasingly popular in the food industry due to their potential health benefits, there has been a growing concern about their allergenicity [215,216]. Although some studies have suggested that bioactive peptides may have reduced allergenicity compared to their parent proteins [217,218], further research is needed to understand their impact on individuals with food allergies fully. However, certain egg proteins, such as OVT, LYS, OVA, and OVM, and EY proteins such as α-livetin and lipoprotein YGP42, are known to cause severe allergy reactions, making it crucial to address their allergenicity [219,220].

Several studies have evaluated the allergenic potential of specific bioactive peptides derived from egg proteins. For example, a study by Rodríguez-Pérez et al. (2017) investigated the allergenicity of a peptide derived from ovalbumin, a major egg white protein, in a mouse model. They found that the peptide had a reduced ability to induce an allergic response compared to the parent protein [221]. In contrast, other studies have shown that certain bioactive peptides may have increased allergenicity compared to their parent proteins. For instance, a study by Chen et al. (2017) found that a peptide derived from ovotransferrin, a major egg white protein, had a higher binding affinity to IgE antibodies than the parent protein, indicating a higher potential for allergenicity [222].

These conflicting results highlight the need for further research to determine the allergenic potential of specific bioactive peptides and to develop standardized methods for allergenicity testing. It is important to conduct proper allergenicity testing for all extracted peptides before use in food products. Heat processing, high-pressure treatment, and enzymatic hydrolysis can induce conformational changes in these proteins, reducing their allergenicity. These methods have been extensively studied to increase the potential of egg proteins as nutraceutical and pharmaceutical agents.

### 4.1. Egg Allergy

The term “food allergy” refers to symptoms and conditions brought on by immunological reactions to food allergens in people already predisposed to having them. IgE antibody production is the most typical immunological response indicative of a food allergy [223,224]. Since an adverse immunological reaction to egg proteins causes atopic dermatitis and eosinophilic esophagitis, it follows that egg allergy is an IgE antibody-mediated allergy [225,226]. Egg proteins’ ability to provoke an immune response is reflected in their stability in the face of heat and enzymatic digestion [206,207,208,209,210,211,212,213,214,215,216,217,218,219,220,221,222,223,224,225,226,227]. While both egg components contain proteins that can trigger an allergic reaction, the allergenic potential of EW proteins is greater than that of EY proteins. The four major proteins present in the EW, which include OVM (Gal d 1), OVA (Gal d 2), OVT (Gal d 3), and LYS (Gal d 4), are the primary culprits. Conversely, recent research suggests that the allergenicity of α-livetin (Gal d 5) and lipoprotein YGP42 (Gal d 6) EY proteins is relatively lower than that of those found in egg white and that they are minor allergens [228].

The EW protein OVM is the allergen that causes the most severe reactions. The disulfide bonds within its three structurally distinct domains (I, II, and III) aid in its resistance to heat denaturation and proteolytic digestion [229,230]. OVM is a serine protease inhibitor that protects against trypsin and elastase, among other proteolytic enzymes [231,232]. Regarding allergenicity, domain III is the most important since it contains both IgE- and IgG-binding epitopes, making it a primary determinant of egg allergy [229,230]. OVM retains its allergenic reactivity even after being subjected to methods that permanently disrupt protein structure, such as heat treatment (e.g., 100 °C for 30 min) or chemical treatment with denaturing detergents (e.g., urea) [233,234]. When compared to OVM, OVA is a low allergen. Six cysteines in the OVA sequence, between Cys74 and Cys121, are connected by disulfide bonds [228]. It belongs to the subfamily of serine protease inhibitors known as clade B serpins. This family of proteins is characterized by the presence of nine α-helices, three β-sheets, and a reactive center loop that is both exposed and mobile and is responsible for their protease inhibitory properties [228]. Minor egg white allergy OVT has a composition of 686 amino acids and 15 disulfide linkages. It is a member of the transferrin family of proteins, which may bind iron. OVT is a soluble glycoprotein with a great deal of similarity to membrane melanotransferrin, both of which suppress iron metabolism and hence belong to the family of transferrins [88]. The IgE reactivity of LYS, an allergen found in egg whites, is low. LYS is relatively resistant to heat denaturation because of its compact structure, which consists of five to seven helices and three-stranded anti-parallel sheets and is held together by four disulfide links [74]. LYS is stable under heat treatment (at 100 °C) for up to 10 min at neutral pH [235].

With 17 disulfide bridges and a single free SH responsible for dimerization, α-livetin is the first allergen discovered among proteins found in EYs. Both egg allergy symptoms and bird-egg syndrome, generated by sensitization to livetin and manifesting as breathing problems, come from exposure to α-livetin [228,236,237]. Although IgE reactivity to the YGP42 protein is the second most common EY allergy, it only occurs in 18% of individuals [238,239]. Despite being heat stable, a modest allergen is broken down by pepsin in the digestive system.

### 4.2. Effect of Processing on the Allergenicity

Proteins are often subjected to different forms of food processing in order to enhance their physicochemical, functional, and nutritional qualities. Proteins’ IgG-binding (antigenic) and IgE-binding (allergenic) characteristics may be affected, changing their allergenic potential. Heat stability, resistance to digestive enzymes, and IgE-binding epitopes on protein molecules are the three main characteristics that influence allergenicity [240,241]. There are two theoretical ways in which processing might modify allergenic properties. Processing can have two effects on proteins’ ability to induce allergic sensitization, which is defined as the specific immunological priming through the intake of proteins. IgE processing can break the integrity of epitopes on the protein structure that are recognized by IgG and IgE antibodies to elicit allergic reactions, and this is the most commonly investigated effect, and processing can reduce the ability of proteins to induce allergic sensitization [242,243]. Thermoprocessing, enzymatic hydrolysis, and high-pressure treatment are the most investigated methods for modifying egg proteins (Table 3).

Alterations to the secondary and tertiary structures of proteins caused by heat treatment may eliminate, disguise, or expose conformational epitopes, all of which profoundly affect allergenicity [244,245]. Furthermore, protein allergenicity may be chemically changed due to interactions between allergenic proteins and other components (such as sugar, oxidized lipids, and polyphenols). For instance, purified OVM showed increased IgE binding ability when heated at 50 °C for 96 h in the presence of glucose. This is because, during this process, a Maillard reaction occurred between the OVM and the glucose, resulting in the modification of lysine and arginine residues through glycation, which may alter the affinity/accessibility of allergens for IgE antibodies [246,247]. Watanabe et al. evaluated OVA-induced allergies in a mouse model by producing IgE in transgenic mice expressing T-cell receptors specific to OVA [228]. OVA was subjected to three distinct heat and pressure combinations: 80 °C for 15 min, 100 °C for 5 min, and 121 °C for 40 min at a pressure of 215,750 Pa. Mice given OVA cooked at 100 °C for 5 min demonstrated a reduction in T-cell responses and an increase in IgE level, indicating the allergenicity of OVA was reduced. Accordingly, the allergenicity of egg proteins may be reduced via heat processing, or novel allergenic compounds can be formed through a Maillard reaction between proteins and reducing sugar [248].

Enzymatic hydrolysis is a superior method to heat processing for reducing the allergenicity of proteins since it may collapse and cleave conformational and linear/sequential epitopes. It can also readily enhance digestibility [249,250]. López-Expósito et al. used pepsin hydrolysis at high pressure (0.4 × 10^9^ Pa) to remove allergenic epitopes from ovalbumin by breaking up the protein sequences [251]. Although certain hydrolysates (e.g., Leu_124_-Phe_134_, Ile_178_-Ala_187_, and Leu_242_-Leu_252_) still retained IgE-binding epitopes, they discovered that other hydrolyzed peptides (e.g., Phe_358_-Phe_366_) only had one IgE-binding site, thereby increasing immune system tolerance to ovalbumin. Additionally, Matsumoto et al. studied the enzymatic hydrolysis of OVA using different proteases. They discovered that alcalase (alkaline protease) from Bacillus licheniformis was the most effective in completely degrading OVA, thereby almost eliminating the allergenic activity of the hydrolysates [252]. In contrast, Jiménez-Saiz et al. used IgE-binding ability and basophil degranulation assays to investigate the immunological activity of digested lysozyme in the presence of simulated gastric and gastroduodenal fluids [253]. Since the lysozyme was only partially degraded in the stomach before being precipitated in the duodenum, it was shown to retain its ability to bind IgE and activate basophils. Fragments 57–83 and fragments 108–122, connected by disulfide bonds, are IgE-binding epitopes, and their release was facilitated by enzymatic digestion. The allergenicity of egg yolk IgY was investigated by Akita et al., who used the passive cutaneous anaphylaxis (PCA) test and enzyme-linked immunosorbent assay to determine its allergenicity. Hydrolysate fragments generated by pepsin digestion were shown to have a decreased IgE antibody response, which was, in turn, linked to either an increase in suppressor T-lymphocytes or a decrease in helper T-lymphocytes [254].

OVM’s structure was altered by transglutaminase such that it was less resistant to digestion by trypsin, as shown by the work of Porta et al. [96]. It has been reported that the Arg_89_-Ala_90_ dipeptide on residues 65–130 of OVM is responsible for trypsin inhibitory activity [255]. However, when OVM was transglutaminase-modified, Gln115 was presented as an acyl donor for transglutaminase to form a covalent monodansylcadaverine conjugate, which reduced the IgE-binding opportunities. Abeyrathne et al. hydrolyzed OVM using several enzyme combinations, including (1) pepsin, (2) alcalase, (3) alcalase + trypsin, and (4) alcalase + papain. They discovered that OVM hydrolyzed with alcalase and trypsin had the best antioxidant activity, whereas OVM hydrolyzed with pepsin had the lowest allergenicity risk and the highest ACE inhibitory action [256]. Using pepsin-coupled Sepharose 4B, OVM was hydrolyzed into three primary fractions with molecular weights of 25,000, 18,000, and 13,000 Da. Hydrolyzed OVM elicited far lower antibody responses than intact OVM, as shown by a positive PCA reaction against no fractions in a partial complement fixation assay, and by using protease and glutathione-Sepharose 4B for enzymatic hydrolysis, Mine et al. also attempted to decrease the allergenicity of egg white proteins, particularly those located on OVM domain III. Initially, two IgE binding sites (e.g., Lys_29_-Ser_44_ and Thr_49_-Cys_56_) were identified on OVM domain III; however, the -helix structure of OVM was disrupted at the location of glycine 32 and phenylalanine 37 by enzymatic hydrolysis, thus breaking the IgE epitopes to reduce the allergenicity [257].

In order to reduce allergenicity, proteins may be subjected to a high-pressure treatment, which is thought to be an efficient processing method for unfolding the protein structure and shifting the location of epitopes on the protein sequence. The high pressure prevents a bridge from forming between two IgE antibodies on the protein and reduces the synthesis of basophil activation mediators [258,259,260]. The allergenicity of OVM was further evaluated by subjecting it to high pressure (0.1 × 10^6^–0.6 × 10^9^ Pa) and measuring the amount of hexosaminidase released from KU812F human pre-basophilic cells. It was discovered that subjecting OVM to pressures between 0.4 × 10^9^ and 0.5 × 10^9^ Pa significantly reduced the release of hexosaminidase. OVM’s allergenicity was partially reduced because, under pressure, its tertiary structure unfolded, exposing hydrophobic cores near the molecule’s surface [261,262]. Acero-Lopez et al. also studied the impact of high-pressure treatment on OVT [263,264]. Treatment of OVT with pressures greater than 0.2 × 10^9^ Pa at pH 8 resulted in a decrease in the number of sulfhydryl groups, which may be predictive of a decrease in allergenic activity, followed by complete protein denaturation at pressures of 0.6 × 10^9^ and 0.7 × 10^9^ Pa, with conformational changes in the helices, sheets, and turns [250]. The degree to which allergenicity is altered and new epitopes are formed depends on the processing technique, the time the food is exposed to it, and the other components present (e.g., sugar). Epitopes on the protein sequence may be destroyed during processing to reduce IgE-binding capacity, and new epitopes can be created to improve IgE-binding capability. Consequently, it is essential to assess the impact of processing on the allergenicity of egg proteins [265,266].

## 5. Bioactive Peptides Derived from Egg Protein

Due to their high nutritional value, regular egg consumption has been associated with various health benefits. Studies have shown that daily egg intake can reduce the risk of developing type 2 diabetes in middle-aged and older Chinese adults. Additionally, egg consumption has been linked to improved cognitive performance in children and adolescents. A review of multiple studies has concluded that consuming eggs regularly does not increase the risk of cardiovascular disease in healthy individuals. Eggs also provide essential nutrients such as choline and vitamin D, which are crucial for overall health and well-being [267,268,269].

The body easily absorbs eggs’ high-quality protein, making them a great food choice for maintaining and building muscle mass. Choline is another essential nutrient found in eggs that is important for brain development and function and has been linked to a reduced risk of cognitive decline and dementia in older adults. Recent studies suggest that eggs may aid in weight loss and management due to their high protein content, which reduces appetite and increases feelings of fullness, and their low glycemic index, which regulates blood sugar levels. However, individuals with high cholesterol levels or a history of cardiovascular disease should consult their doctor before consuming eggs due to their high dietary cholesterol. Therefore, concerns about their high cholesterol and risk of foodborne illness, mainly salmonella, have led to a decline in their consumption in recent years. However, advancements in the food industry have opened up new avenues for utilizing eggs, such as fractionating EW and EY proteins to create functional food products. Another promising approach is the development of bioactive peptides from individual egg proteins using various methods. These bioactive peptides derived from egg proteins have shown great potential as health-promoting agents, which can be used in different food, cosmetic, and pharmaceutical applications [270,271,272,273].

The bioactive peptides can be extracted from various egg proteins, including OVA, OVT, OVC, LYS, and avidin. Each protein has unique peptides with specific biological activities, making them potential candidates for various food, pharmaceutical, and cosmetic applications (Table 4).

Identifying and isolating bioactive peptides from the egg has led to various studies exploring their potential health benefits. One study found that the bioactive peptides in egg white can help lower blood pressure by inhibiting the angiotensin-converting enzyme [63], which is responsible for converting angiotensin I to angiotensin II, a potent vasoconstrictor that increases blood pressure. This effect has been attributed to peptides such as ovokinin, which is formed by the hydrolysis of ovalbumin. Ovokinin, a dipeptide derived from ovalbumin, has been shown to possess antioxidant, anti-inflammatory, antihypertensive, and antimicrobial properties [274,275]. The tripeptide IRW (Ile-Arg-Trp) derived from ovotransferrin has also been found to have an antihypertensive effect, possibly by reducing the activity of the renin-angiotensin system. They can be used to develop functional foods, dietary supplements, and pharmaceuticals to promote human health. Similarly, the peptide YAEERYPIL (Tyr-Ala-Glu-Glu-Arg-Tyr-Pro-Ile-Leu) from ovotransferrin has been found to possess immune-enhancing, antihypertensive, and antiviral properties [276]. These peptides have potential applications in the development of therapeutic agents for infectious diseases and as dietary supplements for immune system support [277]. Other bioactive peptides from the egg have shown potential as antioxidants, antimicrobials, anti-inflammatory agents, and immunomodulatory agents. For example, the peptide RVPSL (Arg-Val-Pro-Ser-Leu) derived from ovotransferrin has been shown to have antioxidant properties, which may protect against oxidative stress and related diseases such as cancer and cardiovascular disease.

The peptide RYIH (Arg-Tyr-Ile-His) from ovomucin has also been found to have antimicrobial properties, particularly against Helicobacter pylori, a bacterium that can cause gastric ulcers and cancer, as well as antitumor, antioxidant, and anticoagulant properties. These peptides have potential applications in developing functional foods, dietary supplements, and therapeutic agents for cancer treatment [89,278]. The peptide LKPTPEGDL (Leu-Lys-Pro-Thr-Pro-Glu-Glu-Asp-Leu) from lysozyme has been shown to have antimicrobial properties, inhibiting the growth of several bacterial strains, such as *Escherichia coli* and *Staphylococcus aureus* [279].

Avidin-derived peptides have been found to possess antitumor, antiviral, and immunomodulatory properties. These peptides have potential applications in the development of therapeutic agents for cancer treatment and as dietary supplements for immune system support [280].

Various methods can extract bioactive peptides from egg proteins, including enzymatic hydrolysis, thermal treatment, and membrane separation. Enzymatic hydrolysis is the most commonly used method, as it allows for the production of specific peptides with targeted biological activities. However, the process may result in structural and nutritional changes in the peptides and potential allergenicity [281,282].

Thermal treatment is a simple and cost-effective method for extracting bioactive peptides but may result in the denaturation of proteins and loss of biological activity. Membrane separation is a promising method for separating specific peptides based on size and charge. However, the process is complex and requires advanced equipment [283].

### 5.1. Enzymatic Hydrolysis

Enzymatic hydrolysis is a process that has been widely used to extract bioactive peptides from various protein sources, including egg proteins. The use of enzymes such as bromelain, chymotrypsin, ficin, papain, pepsin, and trypsin helps break down the protein sequences into peptides of varying molecular weights and individual amino acids [284]. This process is specific and controlled by several factors, including enzyme concentration, pH, temperature, ionic strength, degree of protein denaturation, and mass transfer rate in the enzyme-protein system. During enzymatic hydrolysis, proteins undergo structural changes, resulting in the collapse of quaternary/ternary protein structures and molecular weight reduction, which modify their functional and sensory properties. These changes are essential for generating bioactive peptides, which are inactive within the parent protein sequence but can be released during enzymatic hydrolysis [285,286]. The functional efficacy of a peptide is subject to the impact of both the position and the type of amino acid residues located within the protein structure. Therefore, understanding the structure of the parent protein is crucial to identifying the bioactive peptides that can be generated through enzymatic hydrolysis. The advantages of enzymatic hydrolysis include its specificity, efficiency, and ability to produce a high yield of bioactive peptides. Additionally, it can be easily scaled up for large-scale production. However, enzymatic hydrolysis has some disadvantages, such as its high cost and the need for specialized equipment and expertise [287].

Target peptides in enzymatic hydrolysis include peptides with antihypertensive, antioxidant, and antimicrobial activities, as well as peptides with immunomodulatory and anti-inflammatory properties. One of the most notable bioactive peptides in hydrolyzed egg proteins is ovokinin, which has been linked to the reduction of high blood pressure. Another bioactive peptide, ovomucin, has been found to have a prebiotic effect, promoting the growth of beneficial gut bacteria. Additionally, hydrolyzed egg proteins contain high levels of essential amino acids, making them a complete protein source that can aid muscle building and recovery [288,289,290].

Enzymatic hydrolysis can also cause changes in the structural and nutritional properties of the peptides, leading to alterations in their bioactivity. Furthermore, there is a risk of allergenicity associated with enzymatic hydrolysis, as some people may be allergic to the enzymes used in the process. Another potential risk is contamination with harmful substances, such as heavy metals or bacteria, during production. Therefore, it is crucial to ensure that the hydrolyzed egg proteins are sourced from a reputable supplier and have undergone rigorous testing to ensure their safety [291,292].

#### Efficacy of Hydrolyzed Egg Proteins on Health Benefits

Hydrolyzed egg proteins are a popular protein source commonly used in dietary supplements and functional foods. The hydrolyzation process involves breaking down the protein molecules into smaller fragments, making them more easily digestible and absorbable by the body [293].

Several studies have examined the efficacy of hydrolyzed egg proteins on various health properties, including muscle growth, weight management, and immune function. Hydrolyzed egg proteins have been widely investigated for their anabolic effects on skeletal muscle. Egg white protein is recognized as a high-quality protein source due to its optimal amino acid composition and high bioavailability. Hydrolyzed egg proteins, resulting from the enzymatic hydrolysis of egg white protein, are characterized by smaller peptides, which exhibit faster absorption rates and utilization for muscle protein synthesis. Recent studies have explored the potential impact of hydrolyzed egg proteins on muscle growth and recovery in young and older individuals [294,295]. In a randomized controlled trial, 50 resistance-trained young men were randomly assigned to either a hydrolyzed egg protein or a carbohydrate placebo group. They consumed the supplement before and after resistance training for 8 weeks. The group consuming hydrolyzed egg protein demonstrated significantly improved muscle thickness and strength compared to the placebo group [296]. Another study examined the effects of hydrolyzed egg protein supplementation on muscle mass and strength in older adults. Participants consumed either a placebo or 20 g of hydrolyzed egg protein daily for 12 weeks. The hydrolyzed egg protein group showed significant muscle mass and strength increases compared to the placebo group [297]. A recent meta-analysis analyzed 13 studies investigating the effects of hydrolyzed egg protein supplementation on muscle mass and strength in healthy adults, finding that consuming hydrolyzed egg protein significantly increased muscle mass and strength compared to a placebo [298]. These findings suggest hydrolyzed egg proteins can be a promising dietary supplement for promoting muscle growth and strength in young and older individuals. However, individual results may vary depending on exercise habits, diet, and overall health status. As with any supplement or dietary intervention, it is advisable to consult a healthcare provider before starting regular use [299,300].

Weight management is a crucial component of overall health and well-being, and protein intake is recognized as a critical factor that can impact weight management. Hydrolyzed egg proteins have been the focus of recent scientific research for their potential to aid in weight management due to their protein content [301,302]. A study published in 2019 examined the effects of hydrolyzed egg white protein on appetite and calorie intake in overweight and obese adults. The results showed that consuming hydrolyzed egg white protein significantly decreased calorie intake compared to the control group. Participants who consumed the hydrolyzed egg white protein also reported feeling fuller after meals and had lower levels of the hunger hormone ghrelin than the control group [303]. Another study published in 2020 investigated the effects of consuming hydrolyzed egg white protein on body composition and metabolic health in overweight and obese adults. The study found that hydrolyzed egg white protein consumption significantly reduced body weight, body fat, and waist circumference. Additionally, the participants who consumed the hydrolyzed egg white protein had lower fasting blood glucose, insulin, and cholesterol levels than the control group [304]. A randomized controlled trial published in 2021 explored the effects of consuming hydrolyzed egg white protein on postprandial glucose and insulin responses in healthy adults. The study found hydrolyzed egg white protein consumption led to significantly lower postprandial glucose and insulin responses than the control group. This led to the conclusion that hydrolyzed egg white protein may be an effective dietary tool for managing healthy adults’ blood glucose and insulin levels [305]. In conclusion, these studies suggest that hydrolyzed egg proteins may positively impact weight management, appetite regulation, and metabolic health. However, further research is required to fully comprehend the underlying mechanisms behind these effects and determine the optimal dosages and long-term safety of hydrolyzed egg proteins [78,227,301,306]. Remembering that protein intake should be balanced with other dietary factors, such as fiber and healthy fats, for optimal weight management and overall health.

Moreover, hydrolyzed egg proteins have gained attention due to their potential immune-boosting effects. Studies have indicated that these proteins can positively impact immune function by enhancing the production of immunoglobulins, cytokines, and other immune-related compounds. Recent research has explored the impact of hydrolyzed egg proteins on immune function through various experiments [307]. One study investigated the impact of hydrolyzed egg white protein on immune function in mice. The researchers found that mice consuming a diet containing hydrolyzed egg white protein had increased levels of immune-related compounds, including immunoglobulins and cytokines, compared to a control group [308]. In another study, the effects of hydrolyzed egg protein on immune function in humans were investigated. Researchers found that healthy adults who consumed hydrolyzed egg protein had increased levels of immunoglobulin A in their saliva, an important antibody that helps to protect against infections [309]. A third study examined the impact of a hydrolyzed egg protein supplement on immune function in athletes. Researchers found that athletes consuming the supplement had significantly higher levels of immune-related compounds, including cytokines and white blood cells, than a placebo group [310]. Taken together, these studies suggest that hydrolyzed egg proteins have the potential to enhance immune function by increasing the production of essential immune-related compounds. However, more research is necessary to fully understand the mechanisms underlying these effects and determine the optimal dosage and timing of hydrolyzed egg protein consumption for immune benefits.

It should also be noted that while hydrolyzed egg proteins may have immune-boosting properties, they should not be relied upon as the sole method of preventing or treating infections or other immune-related conditions. A healthy diet, lifestyle, adequate sleep, and good hygiene support immune function. Moreover, individuals with underlying medical conditions or immunodeficiencies must consult a healthcare provider before taking hydrolyzed egg protein supplements.

### 5.2. Thermal Treatment

Thermal treatment involves subjecting egg proteins to high temperatures to induce protein denaturation and break down larger proteins into smaller peptides. The advantages of thermal treatment include its simplicity, low cost, and ability to produce a high yield of bioactive peptides. However, it has some disadvantages, such as its non-specificity and the potential for the loss of bioactivity due to the high temperatures.

Target peptides in thermal treatment include peptides with antioxidant, antimicrobial, and antihypertensive activities, as well as peptides with immunomodulatory and anti-inflammatory properties. Thermal treatment can also cause changes in the peptides’ structural and nutritional properties, leading to their bioactivity alterations. Additionally, thermal treatment has a risk of allergenicity, as some people may be allergic to heat-treated egg proteins [311].

### 5.3. Membrane Separation

Membrane separation involves using a semi-permeable membrane to separate egg proteins based on their molecular weight. This method allows for the isolation of specific peptides with unique biological activities. The advantages of membrane separation include its specificity, efficiency, and ability to produce a high yield of bioactive peptides. However, it has some disadvantages, such as its high cost and the need for specialized equipment and expertise.

Target peptides in membrane separation include peptides with antihypertensive, antioxidant, and antimicrobial activities, as well as peptides with immunomodulatory and anti-inflammatory properties. Membrane separation can also cause changes in the peptides’ structural and nutritional properties, leading to their bioactivity alterations. Additionally, there is a risk of allergenicity associated with membrane separation, as some people may be allergic to the isolated egg peptides [311].

### 5.4. Electrodialysis Method

Electrodialysis is a novel method utilized to isolate and purify specific egg-derived peptides. This method uses an electric field to selectively remove or concentrate ions from a solution [312]. In the case of egg-derived peptides, electrodialysis can be applied to extract and purify specific peptides with desired functional properties selectively. However, isolation and purification of these peptides from egg proteins can be challenging due to their complex molecular structure and other proteins and peptides in the egg matrix. Electrodialysis has emerged as a promising technique for isolating and purifying egg-derived peptides. The process involves the passage of an egg protein solution through a sequence of alternating anion and cation exchange membranes, which selectively remove or concentrate ions based on their charge. This process separates the egg protein solution into multiple fractions, each containing peptides with specific properties.

Several studies have demonstrated the effectiveness of electrodialysis in isolating and purifying specific egg-derived peptides. For example, a study published in 2015 used electrodialysis to extract and purify egg white peptides with antioxidant properties. The study discovered electrodialysis was a reliable technique for isolating specific peptides with desired functional properties. Another study published in 2019 used electrodialysis to isolate and purify egg yolk peptides with anti-inflammatory properties. The study found that electrodialysis resulted in the selective extraction of peptides with specific molecular weights and charge properties, which led to the isolation of peptides with desirable anti-inflammatory activity [313]. Overall, electrodialysis has been demonstrated to be an effective technique for isolating and purifying specific egg-derived peptides with desired functional properties. The technique has several advantages over traditional peptide extraction methods, including increased selectivity, purity, and processing time reduction. However, further research is required to optimize the electrodialysis process and better understand the mechanisms underlying the selective extraction of specific egg-derived peptides [314].

### 5.5. Glycosylation

Glycosylation is a post-translational modification that involves the addition of a carbohydrate moiety to a protein molecule. This modification has been used to isolate and purify specific egg-derived peptides by taking advantage of the differences in the glycosylation patterns of different proteins. Glycosylation can be used to separate proteins based on their charge, size, and shape, and it can be applied to various techniques such as chromatography, electrophoresis, and mass spectrometry [315,316]. One of the advantages of using glycosylation for the isolation and purification of egg-derived peptides is that it allows for the specific targeting of a particular peptide. This means unwanted compounds can be removed during isolation, resulting in a purer product. Additionally, glycosylation can increase the bioactivity and potency of the peptides, enhancing their therapeutic potential. However, glycosylation has some limitations as well [317]. One of the main drawbacks of using glycosylation is that it can be time-consuming and costly, requiring specialized equipment and expertise. Additionally, the glycosylation patterns of proteins can vary depending on several factors, such as the source, preparation, and processing conditions, making it difficult to achieve consistent results. Recent research has focused on optimizing the glycosylation process to minimize these drawbacks and maximize the benefits. Additionally, there is an ongoing investigation into the specific bioactive properties and potential therapeutic applications of glycosylated egg-derived peptides, particularly in areas such as inflammation, oxidative stress, and cardiovascular disease. The glycosylation process generally shows great potential to isolate and purify egg protein-derived peptides that have improved biological activity and therapeutic properties. However, additional investigation is required to fully understand this approach and optimize its efficacy [318,319].

### 5.6. Chromatography

Chromatography is a widely used method for isolating and purifying specific egg-derived peptides. Chromatography techniques can separate peptides based on differences in their physical and chemical properties, such as size, charge, hydrophobicity, and affinity for certain molecules. Commonly used chromatography techniques include ion exchange, size exclusion, and reverse-phase chromatography. Chromatography provides high selectivity, resolution, and purity of target peptides. However, it can be time-consuming and expensive due to the need for specialized equipment and skilled personnel [107]. Recent research in chromatography has focused on developing more efficient and cost-effective techniques, such as monolithic columns and membrane chromatography. Additionally, there has been an interest in combining chromatography with other separation techniques, such as electrodialysis, to enhance the efficiency of peptide purification. Chromatography is a powerful technique that provides high resolution and specificity when isolating and purifying egg-derived peptides. It can be used to obtain highly purified proteins with specific bioactive properties, which can be used to develop novel food products or pharmaceuticals. The main disadvantage of chromatography is its high cost and technical complexity, which limit its use in industrial-scale production. Moreover, chromatography methods can cause protein denaturation or degradation, resulting in the loss of bioactivity. Additionally, some chromatography methods may require harsh solvents or reagents, which can cause environmental concerns [320,321].

## 6. Conclusions

In conclusion, advanced extraction methods hold great potential for improving the functionality of egg-derived peptides while reducing their allergenicity. Techniques such as enzymatic hydrolysis, heat treatment, and glycosylation have been developed to decrease the allergenicity of egg-derived proteins, thus making them safer for consumption by susceptible individuals. Furthermore, advanced extraction methods, including membrane separation, chromatography, and electrodialysis, can isolate and purify specific egg-derived peptides with desired functional properties, enhancing their bioactivity. Enzymatic hydrolysis can also break down polypeptide sequences and produce bioactive peptides with various health benefits. However, optimizing extraction conditions to maximize functionality and allergenicity reduction poses a significant challenge. Therefore, further research is required to optimize extraction conditions and improve the safety and efficacy of egg-derived peptides.

Moreover, the potential of egg proteins as a source of bioactive peptides has been extensively investigated. Egg proteins can be fractionated into egg white and egg yolk, with individual proteins extracted using various techniques such as liquid chromatography, chemical precipitation, ultrafiltration, and electrophoresis. Enzymatic hydrolysis of egg proteins can produce bioactive peptides with various bioactive properties, including anti-oxidant, anti-hypertensive, antimicrobial, and anti-cancer activities. Despite the promising potential of egg-derived peptides in the pharmaceutical and nutraceutical industries, further research is needed to evaluate their therapeutic capabilities and develop them into genuine applications.

The allergenicity of egg proteins is a major limitation of egg consumption among individuals with allergies. The major allergenic proteins in the egg are ovalbumin, ovotransferrin, ovomucin, ovomucoid, and lysozyme in the egg white and lipovitellins, livetins, phosvitin, and LDLs in the egg yolk. Allergic reactions to egg proteins occur when the immune system mistakes these proteins for harmful substances and mounts an immune response. However, various methods, including thermal processing, enzymatic hydrolysis, and high-pressure treatment, can reduce or eliminate allergenicity by destroying allergenic epitopes and decreasing IgE-binding ability. Nonetheless, proper processing conditions must be evaluated to prevent the formation of new allergenic epitopes. In summary, continued research in this area could enhance the safety and functionality of egg-derived peptides for broader utilization in various industries.

## Figures and Tables

**Table 1:** Methods for fractionating the primary proteins in egg whites.

Method	Processing Conditions	Target Protein	% Yield	Advantages	Disadvantages	Efficiency
Precipitation with Ammonium Sulfate	pH 7.0–8.0, 25 °C	Ovalbumin	70–80%	Low cost, Simple procedure	Loss of biological activity, Contamination with other proteins	Low to moderate
Ion-Exchange Chromatography	pH 4.5–5.5, 25 °C	Lysozyme	60–80%	High purity, Large-scale preparation	High cost, Requires specialized equipment	High
Gel-Filtration Chromatography	pH 7.0–8.0, 25 °C	Ovomucin	50–70%	Separation of high molecular weight proteins	Limited resolution for closely related proteins	Moderate
Hydrophobic Interaction Chromatography	pH 5.5–7.0, 25 °C	Ovotransferrin	50–70%	High purity, Mild conditions	Low yield, Requires specialized equipment	Moderate
High Performance Liquid Chromatography (HPLC)	pH 4.0–5.5, 25 °C	Ovomucoid	20–40%	High purity, Separation of closely related proteins	High cost, Requires specialized equipment	High

**Table 2:** Multiple methods for fractionating the primary proteins in egg yolks.

Method	Processing Conditions	Target Protein	Yield (%)	Advantages	Disadvantages	Efficiency
Precipitation with ethanol or ammonium sulfate	pH adjustment, precipitation with ethanol or ammonium sulfate	Lipovitellin	50–60	Simple and low cost	Co-precipitation of other proteins and impurities	Medium
Ion exchange chromatography	Equilibration, loading, washing, and elution with salt gradient	Phosvitin	20–30	High purity	High cost and low yield	High
Hydrophobic interaction chromatography	Equilibration, loading, washing, and elution with salt and hydrophobicity gradients	Livetin	20–30	High purity	High cost and low yield	High
Gel filtration chromatography	Equilibration, loading, washing, and elution based on molecular size	Vitellin	10–20	High purity and gentle separation	Low yield and low resolution	Medium
Ultracentrifugation	Differential centrifugation based on density and size	Phospholipid transfer protein	5–10	High purity and gentle separation	High cost and low yield	Medium

**Table 3:** Effect of processing on the allergenicity of egg proteins.

Processing Method	Effect on Allergenicity	Processing Conditions	Advantages	Disadvantages
Thermal (boiling, baking, frying)	Decreases allergenicity by denaturation of proteins	Varies based on type and duration of heat treatment	Widely used, simple, effective	May affect sensory properties of food, high temperatures may cause advanced glycation end products (AGEs)
High pressure processing (HPP)	Decreases allergenicity by altering protein structure	Pressure up to 900 MPa for 1–10 min	Maintains nutritional and sensory quality of food, no chemicals used	May require specialized equipment, high cost
Pulsed electric fields (PEF)	Decreases allergenicity by altering protein structure	Electric field strength of 20–40 kV/cm for 1–100 µs	Maintains nutritional and sensory quality of food, no chemicals used	May require specialized equipment, limited research on long-term effects
Irradiation	Decreases allergenicity by altering protein structure	Gamma radiation up to 10 kGy or electron beam radiation up to 3.0 MeV	Effective at eliminating bacteria and parasites, no residual chemicals	Negative perception by some consumers, potential for product quality changes
Ultrasound	Decreases allergenicity by altering protein structure	Frequency of 20–100 kHz for 1–20 min	Non-thermal, low energy, no chemicals used	May require specialized equipment, limited research on long-term effects
Enzymatic hydrolysis	Increases or decreases allergenicity depending on degree of hydrolysis	Varies based on type of enzyme and degree of hydrolysis	Can generate bioactive peptides with functional properties	Difficult to control hydrolysis reaction, may generate bitter peptides
Fermentation	Decreases allergenicity by microbial action	Time and temperature vary based on specific fermentation process	Can generate bioactive peptides with functional properties, may enhance nutritional quality	Requires specific microbial strains and conditions, long processing times
Supercritical fluid extraction (SFE)	Reduces allergenicity by removing lipids and altering protein structure	Temperature of 40–60 °C and pressure of 150–400 bar for 1–2 h	No residual chemicals, low energy consumption	May require specialized equipment, limited research on long-term effects
Microwave-assisted processing	Reduces allergenicity by denaturation of proteins	Power level and time vary based on specific process	Rapid processing time, uniform heating, no residual chemicals	May affect sensory properties of food, limited research on long-term effects
Plasma processing	Alters protein structure and reduces allergenicity	Plasma power and treatment time vary based on specific process	No residual chemicals, can be used on a variety of materials	Requires specialized equipment, limited research on long-term effects

**Table 4:** Some examples of peptides with biological activity are extracted from egg proteins.

Egg Protein	Extraction Method	Mechanism & Conditions	Target Proteins	Biological Activity	Peptides	Allergenicity	Advantages	Disadvantages
Ovalbumin	Enzymatic Hydrolysis	Alcalase enzyme at pH 9.5, 60 °C for 4 h	ACE inhibitory peptides	Hypotensive, Hypocholesterolemic	LKALPMHIR, IQWLEPK, YPAL	Low	High yield, specific peptide extraction	May produce bitter peptides
Ovotransferrin	Thermal Extraction	75 °C for 30 min	Lactoferricin B, ovotransferrin-derived peptides	Antimicrobial, Anticancer	IRW, IQW, IQ	Low	Simple method, low cost	Low yield
Ovotransferrin	Enzymatic Hydrolysis	Hydrolysis by trypsin, chymotrypsin, or pepsin at specific pH and temperature	Ovotransferrin	Antimicrobial, immunomodulatory, antioxidant	OV-6, OV-8, OV-16, OV-17	Low	high yield, mild conditions	requires enzyme, time-consuming
Ovomucin	Membrane Extraction	Ultrafiltration, 10–50 kDa cutoff	Ovomucoid-derived peptides	Antioxidant, ACE inhibitory	LK, DPLV	Moderate	Efficient separation, high yield	High cost of equipment
Lysozyme	Enzymatic Hydrolysis	Pepsin at pH 2, 37 °C for 4 h	ACE inhibitory peptides	Hypotensive, Hypocholesterolemic	LPVP, VPP, LPLP	Low	High yield, simple method	Low bioavailability of peptides
Lysozyme	Electrodialysis	Electrodialysis using a cation exchange membrane	Ovalbumin, ovotransferrin, ovomucin	Antimicrobial, immunomodulatory, antioxidant	Lysozyme-derived peptides	No known allergenicity	Scalable process	Expensive equipment and
Phosvitin	Enzymatic Hydrolysis	Alcalase enzyme at pH 9.5, 55 °C for 6 h	Phosvitin-derived peptides	Antioxidant, Anticancer	RGL, LVG	High	High yield, rich in essential amino acids	Strong fishy odor
Phosvitin	Glycosylation	Glycosylation using lactose and α-galactosidase	None specified	Antioxidant, ACE inhibition, antihypertensive	Phosvitin-derived peptides	No known allergenicity	Mild process conditions	Limited yield
Avidin	Thermal Extraction	85 °C for 10 min	Avidin-derived peptides	Antimicrobial, Anticancer	WYVERN, VYP	Low	Simple method, high yield	High risk of biotin deficiency
Avidin	Chromatography	Affinity chromatography using biotin-agarose resin	None specified	Antimicrobial, antitumor	Avidin-derived peptides	No known allergenicity	High purity	Expensive resin
Egg Yolk Proteins	Enzymatic Hydrolysis	Alcalase enzyme at pH 9.0, 55 °C for 5 h	ACE inhibitory peptides	Hypotensive, Hypocholesterolemic	RVPSL, GSPG	Low	High yield, simple method	Low bioavailability of peptides
Egg White Proteins	Membrane Extraction	Ultrafiltration, 3–10 kDa cutoff	Ovotransferrin-derived peptides	Antimicrobial, Anticancer	VVYP, LFR, IQW	Low	Efficient separation, high yield	Limited diversity of peptides
Egg Shell Membrane Proteins	Enzymatic Hydrolysis	Flavourzyme enzyme at pH 7.5, 50 °C for 4 h	Collagen-derived peptides	Anti-inflammatory, Antioxidant	GPAGP, GAAGP	Low	High yield, rich in hydroxyproline	High cost of enzyme

## Data Availability

The data presented in this study are available on request from the corresponding author.
